# Salt-Tolerant Bacteria Support Salinity Stress Mitigating Impact of Arbuscular Mycorrhizal Fungi in Maize (*Zea mays* L.)

**DOI:** 10.3390/microorganisms13061345

**Published:** 2025-06-10

**Authors:** Randa M. Zaki, Aida H. Afify, Eman H. Ashour, Ahmed M. El-Sawah

**Affiliations:** Department of Agricultural Microbiology, Faculty of Agriculture, Mansoura University, Mansoura 35516, Egypt

**Keywords:** arbuscular mycorrhizal fungi, maize, *Stutzerimonas stutzeri*, salinity, plant growth promotion traits

## Abstract

Egypt’s rapid population increase has resulted in higher water demand. It may significantly reduce the amount of water available for agriculture, increasing the chance of using saline water in agriculture. Using saline water certainly poses a major threat to maize growth and may severely affect the growth and productivity of this important crop. Therefore, the aim of this study was to isolate newly native salt-tolerant bacteria from Egyptian saline soils and assess their ability to produce growth-promoting substances under salinity stress, as well as test the mitigating impact of these isolated salt-tolerant bacteria along with arbuscular mycorrhizal fungi (AMF) in maize plants under salinity stress. We isolated ninety-seven salt-tolerant bacterial isolates, and these isolates show a high ability to grow under different concentrations of NaCl. The nine most efficient isolates show a high ability to produce indole acetic acid (IAA), gibberellic acid (GA), P-solubilized exopolysaccharides (EPS), proline, and antioxidants under different NaCl concentrations. Using the 16S rRNA gene, the most effective isolate STB 89 was identified, and its impact, along with AMF, on the growth of salinity-stressed maize was tested in a pot experiment. Our results showed that the growth parameters (shoot length, root length, dry weight, and leaf area), photosynthetic-related pigments (Chlorophyll a, b, and carotenoids), NPK content, and antioxidant enzymes (PPO, POX, and CAT) were improved significantly at *p* ≤ 0.05 due to the bioinoculant applications, while reduced proline accumulation, Na uptake, and the Na^+^/K^+^ ratio in maize plant tissues were observed compared to the control plants. Moreover, the indices of AMF colonization in maize roots and the count of bacteria in the rhizosphere were enhanced due to the bioinoculant applications under salinity stress. In addition, we found that the combined application was more pronounced than the individual application impact. Hence, our results recommended that salt-tolerant bacteria (STB 89) could support salinity, mitigating the impact of AMF in maize plants, as well as allowing better practical techniques for maize cultivation and soil sustainability under salinity stress.

## 1. Introduction

The high population growth in Egypt has led to an increase in crop cultivation to meet the food gap. This has led to increased water consumption, which accounts for roughly 80–85% of water withdrawals for agricultural practices [1]. This percentage can be reduced by using saline water for irrigation, which can be accomplished by alternating or combining saline water with high-quality water sources [2,3]. However, using saline water certainly poses a major threat to plant growth and productivity, in addition to having negative effects on sustainability by increasing soil salinity [4].

Salinity is a major limiting factor in farming ecosystems, which has adversely impacted food security globally [5]. Irrigation with saline water harms plants in several physiological ways, including increased osmotic tension caused by increased salt concentrations in the root zone of plants, which leads to a reduction in water conductivity, soil aeration, and osmotic potential, resulting in physiological dryness and nutritional imbalance [6,7]. Furthermore, plants under salinity over-absorb toxic ions such as Na^+^ and Cl^+^, disrupting ion balance and severely impacting enzyme activity, photosynthesis, and protein synthesis in plant tissues [8]. In addition, salinization increases oxidative stress, which in turn causes severe damage to DNA, protein, and lipids, affecting normal cellular functioning [9].

Several studies have found that inoculation with arbuscular mycorrhizal fungus (AMF) can help plants cope with the effects of salinization [2,10,11,12,13,14,15,16,17]. AMF can form a symbiotic relationship with many terrestrial plant roots, which in turn increase the surface area of the root for nutrient and water uptake [18]. Moreover, AMF can maintain continuous movement of P into the roots under salinity stress, which is critical for vital metabolic processes in plant tissues, by producing phosphatase enzyme, which liberates P from its bound form and increases P availability in the soil, as well as by maintaining an intrinsic phosphate concentration (Pi) within the hyphae through polyphosphate formation [7]. Furthermore, AMF improves K uptake, controls Na^+^ translocation to aboveground parts, and regulates Na^+^ concentrations in plant tissues. This is due to mycorrhizal plants’ ability to sequester Na^+^ into vacuoles or exclude it from the cytosol. AMF also assists the host plant in retracting Na^+^ from the xylem and redirecting it away from photosynthetic tissues and toward the roots [5,7]. Additionally, under salinity stress, mycorrhizal plants accumulate more osmolytes such as proline, polyamines, sugars, organic acids, and amino acids, as well as regulate the antioxidant defense system [19,20,21,22], which helps in osmotic adjustment and the scavenging of reactive oxygen species (ROS) [2]. However, there are several challenges to using AMF in conventional agriculture, such as chemical fertilizers and other agrochemicals, as well as intensive tillage, which all have a negative impact on arbuscular mycorrhizal symbiosis, decreasing root colonization, the spread of intra- and extraradical mycelia, spore germination, and functions [23]. Hence, the utilization of salt-tolerant bacteria (STB) has become an eco-friendly approach for alleviating salinity stress [24,25,26,27,28], and it may support the mitigating impact of AMF on plants, challenging salinity stress [29]. Several mechanisms aid STB directly and indirectly mitigate the negative impacts of salt stress on plants including the production of phytohormones [30,31], ACC deaminase [32], and exopolysaccharides [33]. In addition, inoculation with STB could modulate the physiological process in plant tissues including osmoprotectant accumulation [34,35] and antioxidant production [34,36,37,38,39,40].

Maize (*Zea mays* L.) is considered Egypt’s second most important crop [18], yet maize plants are extremely sensitive to salinity, which is one of the leading causes of plant stress [41]. In this context, our aim was to isolate newly native salt-tolerant bacteria from Egyptian saline soils and assess their ability to produce growth-promoting substances under salinity stress. We hypothesized that inoculation of maize plants with STB would increase the mitigating impact of AMF and assist maize plants in tolerating salinity stress. To test our hypothesis, we tested a variety of morphological, biochemical, physiological, and microbiological characteristics to obtain a deeper understanding of the underlying mechanisms of bioinoculants’ ability to reduce salinity stress in maize plants.

## 2. Materials and Methods

### 2.1. Soil Sampling and Isolation of Salt-Tolerant Bacteria

Soil samples were collected from several locations in Egyptian saline soils, as shown in Figure S1. The samples were taken from a depth of 0–15 cm, stored in an icebox, transferred to the laboratory, and then air-dried and analyzed for electrical conductivity (EC) and pH [42], as presented in Table 1. Pour-plating method was used for bacterial isolation from soil samples using nutrient agar medium (OXOID, Basingstoke Hampshire, UK), and then the picked colonies were maintained on nutrient agar slopes according to the study of Nader et al. [43]. After that, the bacterial isolates were grown on nutrient broth medium (OXOID, Basingstoke Hampshire, UK) containing different concentrations of NaCl (0%, 3%, 6%, 9%, 12%, and 15%). The bacterial cultures were incubated for 72 h at 30 °C, and the optical density (OD) was measured by a spectrophotometer at 600 nm according to the study of Abdelsattar et al. [44].

### 2.2. Estimating Plant Growth Promotion Traits of Salt-Tolerant Bacteria

Briefly, 1 mL of 24 h old culture of salt-tolerant bacterial isolates was inoculated in 100 mL of nutrient broth medium in conical flasks (250 mL). Incubation of the bacterial cultures was performed at 30 °C on a rotary shaker (150 rpm). To induce salinity stress, different concentrations of NaCl (0%, 3%, 6%, 9%, 12%, and 15%) were supplied to the liquid media. For indole acetic acid (IAA) measurement, 0.1% tryptophan was added to the liquid media, and IAA was assayed calorimetrically in the culture supernatant at 530 nm after 2 days of incubation [45]. Gibberellic acid (GA) was determined calorimetrically at 760 nm [46]. Pikovskaya medium [47] was used to determine the ability of isolated bacteria to solubilize tri-calcium phosphate, and the P-solubilized was determined in the supernatant at 660 nm using a spectrophotometer (Thermo Scientific BioMate 3, Thermo Electron Corporation, Waltham, MA, USA) [48,49]. Exopolysaccharides (EPS) were determined as in the study of Sharath et al. [50] after 3 days of incubation, and calculated as mg/L. Proline was determined using the ninhydrin colorimetric method at 520 nm in the culture supernatant [51]. Free radical scavenging assay was estimated as a non-enzymatic antioxidant assay using the protocol described by Abou-Aly et al. [46].

### 2.3. Identification of Bacteria

Bacterial identification was performed by the National Research Centre (NRC), Egypt, using 16S rRNA sequencing. The BLAST algorithm (https://blast.ncbi.nlm.nih.gov/Blast.cgi, accessed on 31 May 2025) was used to compare the acquired sequences to the known sequences in the NCBI database. Aligning 16S rRNA sequences was performed using MEGA 12.0 software. The bacterial accession number for the 16S rRNA sequence was obtained from the GenBank of NCBI.

### 2.4. Experimental Setup

A pot experiment was carried out during the summer season of 2024 at the experimental farm of the Faculty of Agriculture, Mansoura University, Egypt (27.00° N, 30.00° E). Plastic pots of 25 cm (diameter) and 30 cm (height) were filled with 10 kg pot^−1^, and the experimental soil’s physical, chemical, and biological characteristics are presented in Table 2. The experiment included 36 pots that were arranged in a complete randomized block design with four experimental settings as follows: (1) Control; (2) AMF; (3) STB; and (4) AMF + STB. Irrigation was performed with three concentrations of saline water (CK, 50 mM NaCl, and 100 mM NaCl). For the AMF application, 5 g of trapped soil (50 spores g^−1^ soil) and 0.5 g of fresh Sudan grass roots colonized with *Glomus clarum*, *Funneliformis mosseae*, and *Gigaspora margarita* (with 70% colonization) were added to each pot. To produce the mycorrhizal plants, the inoculum was placed roughly 5 cm below the soil surface to encourage fungal colonization of maize roots. An equal amount of autoclaved soil and colonized root inoculum was added to the non-mycorrhizal plants to maintain comparable nutritional conditions. For bacterial application, the seeds were immersed in a liquid culture of *Stutzerimonas stutzeri* PV248835 (STB 89) previously grown on nutrient broth medium for 2 days at 30 °C (10^8^ cfu/mL) for 30 min before sowing. Arabic gum (16%) was used as an adhesive agent. After 14 days since sowing, each plant was inoculated with an extra 10 mL of the bacterial culture. The non-bacterial treatments received an equal volume of autoclaved inoculum. Maize seeds (Triple Hybrid, Hoth 352) were planted (10 seeds/pot) after sterilization with NaClO 0.5% for five minutes, and the plants were thinned to three plants per pot.

### 2.5. Bacterial Count Determination and Estimation of Mycorrhizal Colonization

The pour-plating method was used for bacterial count in the rhizosphere of maize plants. The total bacterial count (TBC), phosphate-solubilizing bacterial count (PSBC), and potassium-releasing bacterial count (KRBC) were counted according to the study of Gao et al. [18] on nutrient agar medium [52], Pikovskaya’s medium [47], and Alexandrov’s medium [53], respectively. The mycorrhizal colonization was determined in trypan-blue-stained maize roots according to Phillips and Hyman [54], and then the mycorrhizal indices (frequency, F%; intensity, M%; arbuscule frequency, A%) were determined using the Mycocalc program (https://www2.dijon.inrae.fr/mychintec/Mycocalc-prg/download.html, accessed on 15 March 2025) according to Trouvelot et al. [55].

### 2.6. Growth Measurements and Photosynthetic Pigments Determination

After 60 days, 3 plants from each treatment were randomly selected. Then, shoot length, root length, shoot dry weight, and root dry weight according to the study of Gao et al. [18]. Leaf area was measured in the mature leaf in the middle quadrant of the plant according to the method of Anmaullah et al. [56] by using the following formula: LAI = L × W × K (where L = leaf length, W = leaf width, and K = constant ≈ 0.75). Photosynthetic pigments were determined according to the method of Lichtenthaler and Wellburn [57], briefly, fresh leaf samples (0.2 g) were homogenized in 3 mL of ethanol (95%, *v*/*v*), and leaf chlorophylls a, b, and carotenoids were determined spectrophotometry at 665, 649, and 470 nm, then their concentrations were calculated in mgL^−1^.

### 2.7. Determination of Nutrients, Proline, and Enzyme Activities

Total N and P contents were determined using the methods of Kjeldhal (Behr distillation unit, Germany) [42] and Snell and Snell [58], respectively. K and Na contents were determined by atomic absorption spectroscopy (GBC Scientific Equipment model SENSAA Dual, Australia) [59]. Proline was determined in the fresh leaf samples using the ninhydrin colorimetric method, and its concentration was calculated and expressed as µg^−1^ FW according to Bates et al. [51]. Polyphenol oxidase (PPO), Peroxidase (POX), and Catalase (CAT) activities were measured at 495 nm, 470 nm, and 240 nm by tracking the changes in absorbance every 30 s intervals for 3 min, and the enzyme activities were expressed as U g^−1^ FW, following [60,61,62], respectively.

### 2.8. Statistical Analysis

The data presented are the means ± standard deviation (SD). Using SPSS software version 27.0, a one-way analysis of variance ANOVA and Duncan’s multiple range test at the 0.05 level was performed. The software of CoStat was used to evaluate the interaction between bio-inoculation and salinity stress using two-way ANOVA, and * *p* < 0.05, ** *p* < 0.001, and *** *p* < 0.0001 indicate significant differences between factors. RStudio Version 4.5.0 was used to perform the principal component analysis and the heatmap of correlation. The presentation of graphs was performed using OriginPro 2024 software.

## 3. Results

### 3.1. Isolation and Screening of Salt-Tolerant Bacteria

Data regarding some chemical properties of the collected soil samples from different locations, including Klabsho, North Delta, Bahariya Oasis, El-Senbellawein, and Gamasa, are presented in Table 1. The electrical conductivity (dSm^−1^) of the studied soil samples S1, S2, S3, S4, S5, S6, S7, S8, S9, with S10 being 10.25, 2.63, 3.38, 2.38, 9.90, 10.71, 7.19, 9.35, 7.36, and 2.11, respectively. However, pH values ranged from 7.98 to 8.79. Ninety-seven bacterial isolates were isolated from nutrient agar plates and evaluated for growth in various NaCl concentrations (0%, 3%, 6%, 9%, 12%, and 15%), as shown in Tables S1A–D. Nine bacterial isolates (STB 8, STB 30, STB 38, STB 41, STB 59, STB 60, STB 77, STB 88, and STB 89) were chosen for further studies due to their high ability to grow under salinity stress as calculated by (increase/decrease) percent (%) of growth compared to the control (Tables S2A–D).

### 3.2. Plant Growth Promotion Traits of Salt-Tolerant Bacteria

**Figure 1:**
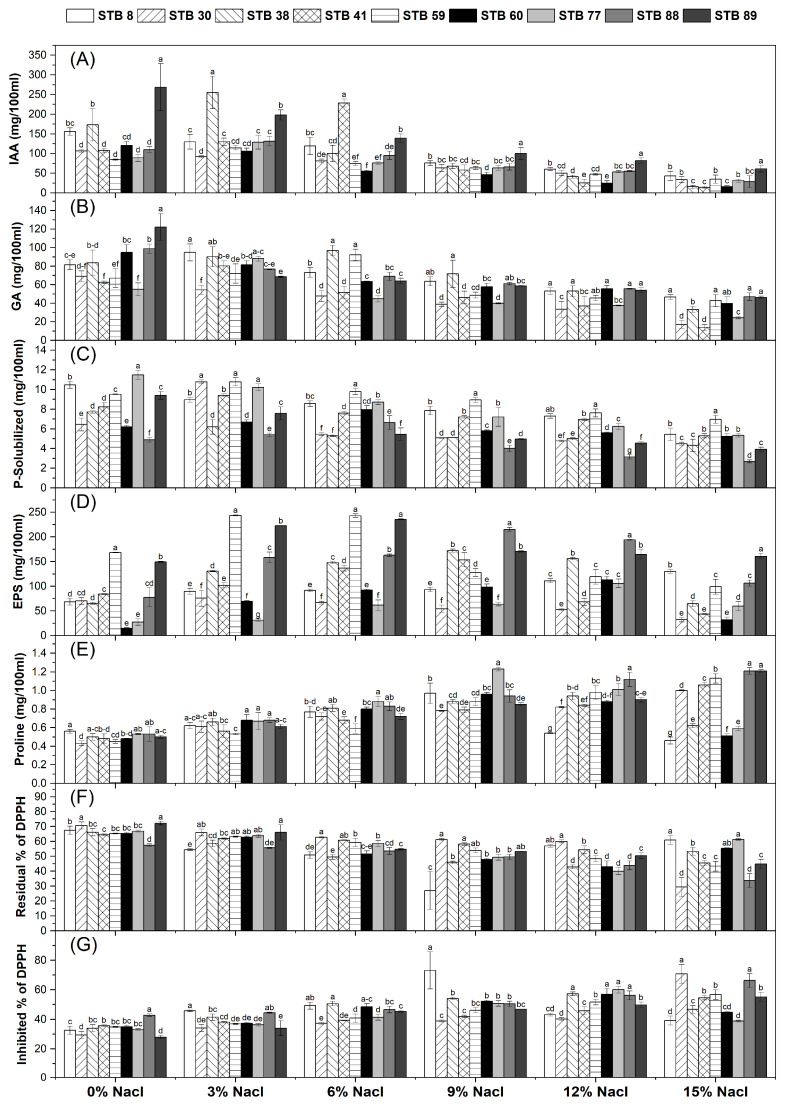
Plant growth promotion traits of salt-tolerant bacterial isolates. (**A**), IAA; (**B**), GA; (**C**), P-Solubilized; (**D**), EPS; (**E**), Proline; (**F**), Residual % of DPPH; (**G**), Inhibited % of DPPH. Data are means ± standard deviation; different letters within the same group indicate significant differences between means at *p* ≤ 0.05. IAA, Indole acetic acid; GA, Gibberellic acid; EPS, Exopolysaccharides.

The high-tolerant nine isolates were tested to produce IAA, GA, P-solubilization, EPS, proline, and antioxidants with various concentrations of NaCl (0%, 3%, 6%, 9%, 12%, and 15%). The results in Figure 1A showed that all the tested isolates could produce IAA at various concentrations of NaCl (0%, 3%, 6%, 9%, 12%, and 15%); however, the IAA production decreased as NaCl concentration increased in the culture. The maximum production of IAA was recorded for isolate STB 89 (269.56 mg/100 mL), which steadily reduced as the NaCl concentration increased until reaching 61.12 mg/100 mL at 15% NaCl. A similar trend was observed in GA production, which decreased as NaCl concentration increased in the culture (Figure 1B). The maximum production of GA was recorded for isolate STB 89 (122.02 mg/100 mL), which steadily reduced as the NaCl concentration increased until reaching (45.12 mg/100 mL) at 15% NaCl, followed by isolate STB 88 (98.81 mg/100 mL), which steadily reduced as the NaCl concentration increased until reaching (55.57 mg/100 mL) at 15% NaCl. For P-solubilization, all the tested isolates have the ability to solubilize P at various concentrations of NaCl (0%, 3%, 6%, 9%, 12%, and 15%). The highest P-solubilized amounts (11.48, 10.47, 9.51, and 9.40 mg/100 mL) were recorded for isolates STB 77, STB 8, STB 59, and STB 89, respectively (Figure 1C).

Regarding exopolysaccharides production (EPS), all the tested isolates have the ability to produce EPS at various concentrations of NaCl (0%, 3%, 6%, 9%, 12%, and 15%); however, the EPS production increased as NaCl concentration increased in the culture (Figure 1D). The highest EPS production was recorded for isolates STB 59 and STB 89 (168.20 and 149.27 mg/100 mL). The same pattern was observed for proline production, with the highest amounts (0.56, 0.53, 0.53, 0.50, and 0.50 mg/100 mL) for isolates STB 8, STB 77, STB 88, STB 38, and STB 89, respectively (Figure 1E).

Data in Figure 1F indicate that the maximum residual DPPH (minimum inhibition) by the bacterial isolates was observed in cultures without NaCl. However, the inhibition of DPPH by the bacterial isolates generally increased with the increasing concentration. The maximum inhibition of DPPH was recorded in the culture at 9% NaCl (73.09%) by STB 8, followed by isolates STB 30 and STB 88 at 15% NaCl (70.73 and 66.24%), respectively (Figure 1G).

### 3.3. Identification of the Most Effective Salt-Tolerant Bacterial Isolate

Salt-tolerant bacterial isolate (STB 89) was chosen for identification because it could produce substantial amounts of plant growth-promoting substances under salinity stress conditions, as previously reported. The bacterial isolate was identified as *Stutzerimonas stutzeri* according to the NCBI database. Accession number PV248835 was deposited for the bacterial isolate in NCBI GenBank (Figure S2).

### 3.4. Bacterial Count and Mycorrhizal Colonization Under Salinity Stress

The effect of bioinoculant applications on the bacterial count in the rhizosphere of maize plants under either control or salinity stress conditions is presented in Table 3. Under both unstressed and stressed plants, the bacterial count in the rhizosphere of bioinoculant-inoculated treatments was higher than that of uninoculated treatments. Furthermore, as the concentration of salinity increased, the bacterial count (TCB, PSBC, and KRBC) decreased. Considering the two salinity levels (50 mM NaCl and 100 mM NaCl), the average decrease in the TCB, PSBC, and KRBC was 5.73, 2.18, and 1.44%, respectively, as compared with the control. However, the bioinoculant applications increased the bacterial counts under saline stress compared with their corresponding control. Also, the combined treatment at different salinity concentrations gave the highest bacterial counts. In addition, it was observed that inoculation with STB enhanced total bacterial count more than AMF. The total bacterial count was improved with the application of AMF, STB 89, and combination treatments by (3.24, 5.32, and 7.47%) in the rhizosphere of treated plants compared with the untreated control plants. A similar direction was noted for phosphate-solubilizing bacterial count and potassium-releasing bacterial count (Table 2). Phosphate-solubilizing bacterial count and potassium-releasing bacterial count were improved with the application of AMF, STB 89, and combination treatments by (2.40, 4.04, and 6.07%) and (0.97, 2.64, and 3.17%), respectively, in the rhizosphere of treated plants compared with the untreated control plants.

**Figure 2:**
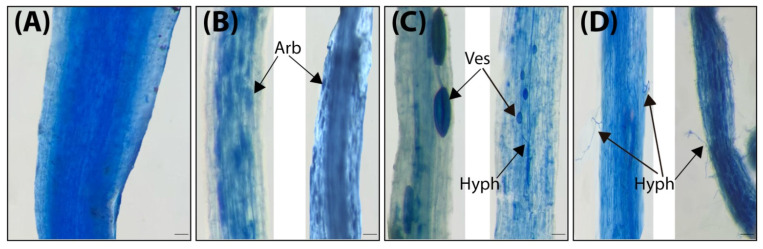
Photomicrographs of maize root cells (Scale bars represent = 20 µm): (**A**), control without colonization; (**B**–**D**), AMF-inoculated maize roots showing mycorrhizal structure; Arb, Arbscules; Ves, Vesicles; Hyph, Hyphae.

The frequency (F%), intensity (M%), and arbuscular development (A%) of mycorrhizal colonization in the roots of maize plants were measured (Table 2). Regardless of salinity stress, the data revealed that the mycorrhizal colonization in maize roots was increased with the combined application when compared to the single application of AMF. However, salinity stress significantly altered the indices (F, M, and A%) of AMF colonization in maize roots, and there were significant interactions between salinity stress and mycorrhizal colonization. Also, the mycorrhizal colonization gradually declined as the concentrations of salinity increased. In addition, there was no mycorrhizal colonization of non-mycorrhizal plant roots. Figure 2 shows the mycorrhizal structures in the stained roots of maize plants. These findings support AMF’s ability to colonize maize roots at all salinity levels.

### 3.5. Morphological Traits of Maize Plants Under Salinity Stress

Mean data regarding the morphological response of maize plants grown under either control or salinity stress conditions are presented in Table 4. The data reveal that salinity stress significantly reduced plant growth and biomass accumulation compared with the corresponding control maize plants. Considering the two salinity levels (50 mM NaCl and 100 mM NaCl), the average decrease in the shoot length, root length, SDW, RDW, and leaf area was 27.50, 22.21, 45.46, 52.98, and 31.11%, respectively, as compared with the control plants. Irrespective of the salinity stress effects, AMF, STB, and their combination treatments improved plant growth and biomass of maize plants compared with control plants. In general, the highest values of plant growth and biomass were observed in plants treated with the combined application. Shoot and root lengths improved with the application of AMF, STB 89, and their combination treatments by (18.03, 11.24, and 23.31%) and (26.02, 19.55, and 35.39%), respectively, compared with the control plants. Furthermore, the dry weight of shoots and roots was improved with the application of AMF, STB 89, and their combination treatments by (38.58, 24.67, and 50.18%) and (63.60, 45.33, and 74.45%), respectively, compared with the control plants. In addition, leaf area was improved with the application of AMF, STB 89, and their combination treatments by (32.64, 24.33, and 42.72%), respectively, compared with the control plants. These results indicated that the morphological traits of maize plants were enhanced significantly under salinity stress due to the bioinoculant applications.

### 3.6. Photosynthetic-Related Pigments of Maize Plants Under Salinity Stress

**Figure 3:**
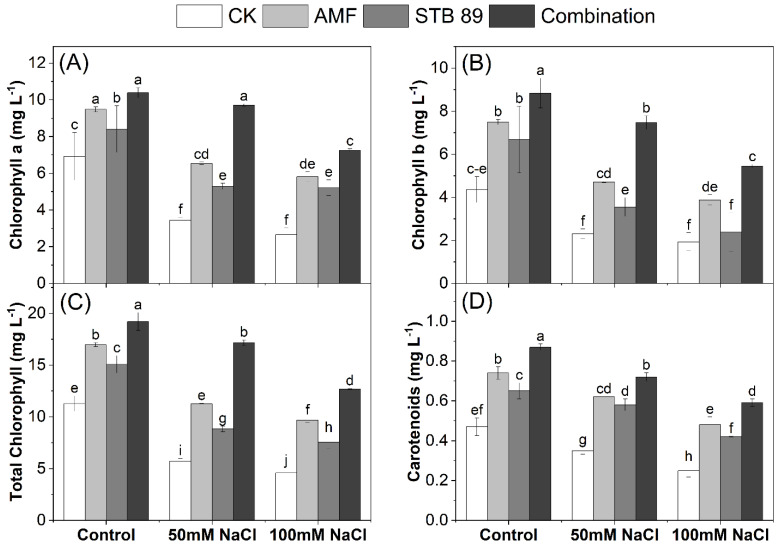
Changes in photosynthetic-related pigments in the leaves of maize plants grown under either control or salinity stress conditions: (**A**): chlorophyll a, (**B**): chlorophyll b, (**C**): total chlorophyll, (**D**): carotenoids. Data are means ± standard deviation; different letters indicate significant differences between means at *p* ≤ 0.05.

Although chlorophyll is essential for keeping plants green and healthy, salinity reduces chlorophyll content in salt-sensitive plants. Bioinoculants could be a promising strategy to improve photosynthetic apparatus under salinity stress. Mean data regarding chlorophyll contents in the leaves of maize plants grown under either control or salinity stress conditions are presented in Figure 3. Chlorophyll content was decreased as the concentration of salinity level increased. Considering the two salinity levels (50 mM NaCl and 100 mM NaCl), the average decrease in chlorophyll a, b, total, and carotenoid content in the leaves was 55.92, 51.49, 54.21, and 36.17%, respectively, as compared with the control plants. On the other side, plants treated with the combined application had the highest chlorophyll content, followed by AMF and STB 89 applications, under either control or salinity stress conditions. Inoculation with AMF, STB 89, and their combination resulted in an increase in chlorophyll a, b, total, and carotenoids content in the leaves by (40.35, 31.14, and 52.37%), (46.61, 31.93, and 60.50%), (43.01, 31.41, and 55.96%), and (41.84, 35.15, and 50.91%), respectively, compared with the control plants. The obtained results reported that the photosynthetic-related pigments in the leaves of maize plants were significantly enhanced under salinity stress due to the bioinoculants application.

### 3.7. Nutrient Content and Determination of the Na^+^/K^+^ Ratio

**Figure 4:**
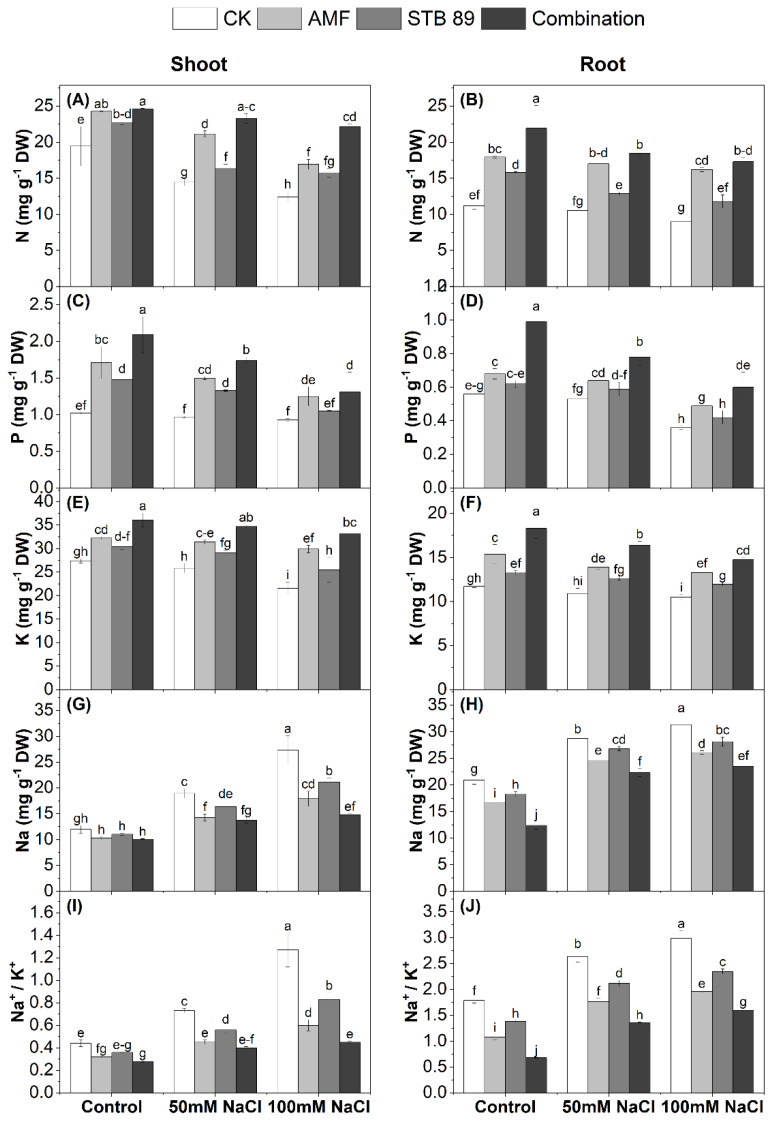
N, P, K, and Na contents (mg g^−1^ DW) and Na^+^/K^+^ ratio in the shoots and roots of maize plants grown under either control or salinity stress conditions. (**A**), N-shoot; (**B**), N-root; (**C**), P-shoot; (**D**), P-root; (**E**), K-shoot; (**F**), K-root; (**G**), Na-shoot; (**H**), Na-root; (**I**) Na^+^/K^+^-shoot; (**J**), Na^+^/K^+^-root. Data are means ± standard deviation; different letters indicate significant differences between means at *p* ≤ 0.05. Ck, Check; AMF, Arbuscular mycorrhizal fungi; STB, Salinity tolerant bacteria “*Stutzerimonas stutzeri* PV248835”.

Mean data regarding N, P, K, Na contents, and the Na^+^/K^+^ ratio in the leaves and roots of maize plants grown under either control or salinity stress conditions, are presented in Figure 4. The contents of N, P, and K decreased significantly as the concentration of salinity increased. Considering the two salinity levels (50 mM NaCl and 100 mM NaCl), the average decrease in N, P, and K in the leaves and roots was (30.88, 6.86, and 13.58%) and (12.92, 20.53, and 8.69%), respectively, as compared with the control plants. Compared with the corresponding control plants, inoculation with AMF, STB 89, and their combination increased N content in maize shoots and roots by (25.66, 15.40, and 33.77%) and (39.89, 23.97, and 46.71%), respectively (Figure 4A,B). Furthermore, P content was increased by (34.52, 24.35, and 43.19%) and (19.88, 11.04, and 38.81%), respectively (Figure 4C,D). In addition, K content was increased by (20.00, 12.04, and 28.07%) and (22.14, 12.46, and 32.98%), respectively (Figure 4E,F). On the contrary, there was an increase in Na content and the Na^+^/K^+^ ratio as the concentration of salinity increased. Considering the two salinity levels (50 mM NaCl and 100 mM NaCl), the average increase in Na content and the Na+/K+ ratio in the leaves and roots was (46.47 and 52.54%) and (30.18 and 36.16%), respectively, as compared with the control plants. However, inoculation with AMF, STB 89, and their combination could decrease Na uptake by (27.17, 16.61, and 33.62%) and (16.90, 9.56, and 28.12%) in the shoots and roots, respectively (Figure 4G,H). In addition, the Na^+^/K^+^ ratio was increased in the shoots and roots by (43.85, 28.27, and 53.68%) and (35.17, 21.15, and 51.07%), respectively, after inoculation with AMF, STB 89, and their combination. Such results indicate the potential impact of bioinoculants to enhance nutrient uptake and to reduce Na uptake under salinity stress.

### 3.8. Proline Content and Determination of Antioxidant Enzyme Activities

**Figure 5:**
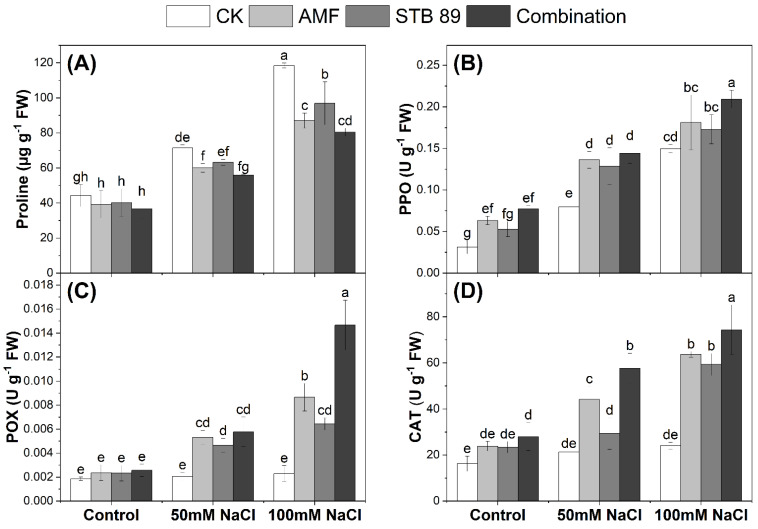
Proline content (µg g^−1^ FW) and antioxidant enzymes activity (U g^−1^ FW) in the leaves of maize plants grown under either control or salinity stress conditions. (**A**) Proline content; (**B**) PPO activity; (**C**) POX activity; (**D**) CAT activity. Data are means ± standard deviation; different letters indicate significant differences between means at *p* ≤ 0.05. Ck, Check; AMF, Arbuscular mycorrhizal fungi; STB, Salinity tolerant bacteria “*Stutzerimonas stutzeri* PV248835”.

Mean data regarding proline contents and the activity of antioxidant enzymes in the leaves of maize plants grown under either control or salinity stress conditions are presented in Figure 5. As expected, the contents of proline were increased as the concentration of salinity increased. Considering the two salinity levels (50 mM NaCl and 100 mM NaCl), the average increase in proline content in maize leaves was 50.28% as compared with the control plants. However, inoculation with AMF, STB 89, and their combination decreased the percentages of proline by (20.42, 14.39, and 26.99%), respectively. Results showed that the combined application reduced proline content more pronouncedly than the sole application of AMF and STB 89. Regardless of the bioinoculants application, the PPO activity increased in the leaves of maize plants as the concentration of salinity increased. However, inoculation with AMF, STB 89, and their combination resulted in a significant increase in PPO activity, and the enzyme activity was increased along with the increase in the concentration of salinity. Maximum PPO activity was observed in the combined application under 100 mM NaCl (Figure 5B). For the activities of POX and CAT, regardless of the bioinoculants application, a slight insignificant increase was observed as the concentration of salinity increased. However, inoculation with AMF, STB 89, and their combination resulted in a significant increase in POX and CAT activities, and the enzyme activities were increased along with the increase in the concentration of salinity. Maximum POX and CAT activities were observed in the combined application under 100 mM NaCl (Figure 5C,D). The results showed that the bioinoculant application had the ability to reduce proline content while also activating the antioxidant enzyme defense system in maize plants under salinity stress, and this response was more pronounced in the combined application than in the individual AMF and STB 89 applications.

### 3.9. Evaluation of the Impact of AMF, STB, and Their Combination by Principal Component Analysis and Heatmap of Correlation Under Salinity Stress

**Figure 6:**
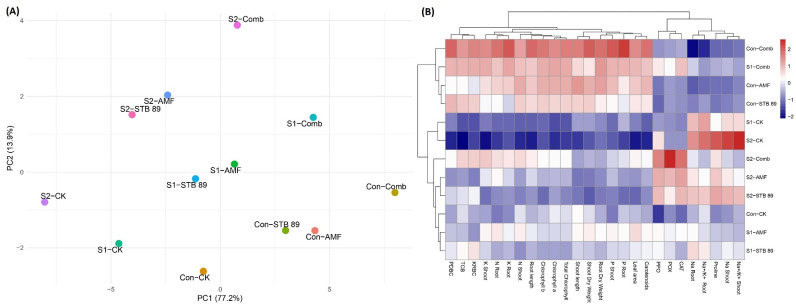
Changes in maize growth-related parameters as affected by AMF, STB 89, and their combination under both normal and salinity stress conditions using multivariate statistical analysis: (**A**) Principal component analysis (PCA). (**B**) Heatmap of correlation.

The maize growth-related parameters were analyzed by PCA (Figure 6A). Two components (PC1 and PC2) were shown from the PCA of the studied traits that explained 77.20% and 13.9% of the data variability, respectively. In addition, hierarchical clustering analysis showed differences in growth-related parameter values in bioinoculants-treated maize plants under control and salinity stress conditions (Figure 6B). These results indicate that bioinoculants contributed significantly to mitigating the detrimental impacts of salt stress on maize plants.

## 4. Discussion

### 4.1. Isolated Salt-Tolerant Bacteria Produce Plant Growth Substances Actively

We isolated a total of 97 bacterial isolates from Egyptian saline soils. All of them could grow in different concentrations of NaCl actively (Table S1A–D). The results showed that all isolates could grow at all salinity concentrations at varying degrees, with nine potent isolates, which were selected due to their high ability to grow under salinity stress conditions (Table S2A–D). The nine most tolerant isolates were screened to produce IAA, GA, P-solubilization, EPS, proline, and antioxidants at various concentrations of NaCl (0%, 3%, 6%, 9%, 12%, and 15%), and all of them could produce these growth substances under both conditions. These compounds may provide additional benefits to plant growth, quality, stress tolerance, and plant health against pathogens [63,64,65,66,67]. Under the salinity stress conditions, the production of IAA, GA, and P-solubilized was decreased as NaCl concentration increased in the culture (Figure 1A–C). However, the production of proline, EPS, and antioxidants was increased as NaCl concentration increased in the culture (Figure 1D–G). The isolate STB 89 was chosen due to high production of plant growth promotion substances under both unstressed and stressed conditions, and this isolate was identified as *Stutzerimonas stutzeri* PV248835. Similarly, Abdelsattar et al. [44] isolated several salt-tolerant bacteria from Egyptian saline soils and found that these bacteria showed a high production of PGP, such as high salt tolerance, N-fixation, P-solubilization, and IAA production. Also, Kumar et al. [68] found that the amount of IAA and P-solubilized produced by salt-tolerant *Bacillus pumilus* JPVS11 gradually decreased as NaCl concentrations increased; however, the proline content was increased with increasing NaCl concentration. Also, Shahid et al. [69] found a higher number of EPS under salt-stress conditions compared with the control. This increase might suggest that bacteria produce proline, EPS, and antioxidants in response to salinity stress. Therefore, the isolated bacteria’s ability to produce growth-promoting traits could help maize plants withstand salt stress.

### 4.2. AMF and STB 89 Improve Bacterial Counts and Mycorrhizal Colonization

We observed an increase in bacterial counts (TCB, PSBC, and KRBC) due to the applications of bioinoculants when compared to the control treatments under both unstressed and stressed plants (Table 3). Such an increase in bacterial count may be attributed to the survivability of nutrients and organic matter, which operate as an energy source for bacterial reproduction, as well as the heat that is produced during OM decomposition, which promotes bacterial growth [18]. Similar observations by Gao et al. [18] found an increase in TCB, PSBC, and KRBC in maize rhizosphere after inoculation with bioinoculants. These results show that the bioinoculants could contribute to helping maize plants absorb more nutrients, particularly P and K, from soil under salinity stress.

Mycorrhizal colonization levels (F%, M%, and A%) were decreased due to salinity stress; however, the used mycorrhizal species could still colonize maize roots. The decrease in fungal development and structure as salinity concentration increased could be ascribed to salt stress, which limits fungal growth and structure. Similarly, both [70] and [2] observed a decrease in fungal growth and spore germination. However, maize plant development improved across all salinity levels, despite the decrease in root colonization, indicating the beneficial impacts of AMF on the host plant growth. The combined treatment had the highest mycorrhizal colonization values under both unstressed and stressed plants (Table 3). Our findings indicated that the AMF strains used can tolerate and form a symbiotic relationship with maize plants and modify the root architecture under high salinity stress, potentially allowing maize plants to absorb more water and nutrients, in particular phosphorus.

### 4.3. AMF and STB 89 Improve the Morphological Traits of Maize Plants

The morphological traits of maize plants have been negatively affected by salinity stress; however, using bioinoculants improved maize growth (shoot length, root length, dry weight, and leaf area) under both unstressed and stressed plants (Table 4, Figure 6A,B). This could be attributed to the capability of *Stutzerimonas stutzeri* PV248835 to produce high amounts of growth-promoting substances (Figure 1), which could explain the improvement in the growth of maize plants. In addition, AMF symbiosis improves plant growth by increasing water availability using hyphae and the concentration of nutrients, particularly P, in plant tissues under salinity. The highest values of morphological traits were observed in the combined treatment under both unstressed and stressed plants (Table 4); this could be attributed to the synergistic effect between bacteria and AMF [10,15,71]. Our results are in line with recent findings, which report that the use of *Funneliformis constrictum* significantly increased the growth of maize under salinity stress [2]. As well as this, de Carvalho Neta et al. [15] found that co-inoculation with AMF and PGPB was more pronounced in improving maize growth under salinity stress rather than individual inoculation. Our findings indicate that inoculation with *Stutzerimonas stutzeri* PV248835 could help improve the impact of AMF on maize growth under salinity stress.

### 4.4. AMF and STB 89 Improve Photosynthetic-Related Pigments Content in Maize Plants

Although chlorophyll is essential for keeping plants green and healthy, salinity reduces chlorophyll content in salt-sensitive plants. Such a decrease may be attributed to salt stress, which accelerated the degradation of chlorophyll, especially Chlorophyll b, which is an important component of the PSII light-harvesting pigment protein complex, and its reduction would directly damage the structure and function of this complex. Our results indicate that bioinoculants could be a promising strategy to improve photosynthetic apparatus under salinity stress (Figure 3), as the used bioinoculants (AMF, STB 89, and their combination) could aid maize plants in increasing chlorophyll content under all levels of salinity stress. Previous studies reported the beneficial impact of AMF on enhancing photosynthetic pigments under salinity stress; likewise, El-Sawah et al. [2] found a significant increase in photosynthetic pigment contents in the leaves of maize under salinity stress after inoculation with *Funneliformis constrictum*. However, our results indicate that inoculation with *Stutzerimonas stutzeri* PV248835 in combination with AMF could strengthen their impact on photosynthetic pigment contents, and thus maize growth.

### 4.5. AMF and STB 89 Enhance Nutrient Content and Reduce the Na^+^/K^+^ Ratio in Maize Plants

Salinity increased the absorption of Na^+^, whereas it decreased N, P, and K uptake, and also increased the Na^+^/K^+^ ratio (Figure 4). However, the obtained results showed that AMF, STB 89, and their combination could enhance N, P, and K uptake; also, a decrease was observed in Na^+^ and the Na^+^/K^+^ ratio under salinity stress compared to the control plants. That impact could be attributable to the AMF and beneficial bacteria providing P nutrition to host plants [18] and compartmentalization of Na^+^ ions [16]. Also, AMF can restrict the transport of Na from roots to shoots in plants, which decreases Na^+^/K^+^ in leaves and stems, in addition to protecting the photosynthetic organs from damage [16,72]. Similar studies found that inoculation with AMF and/or PGPR reduces the accumulation of Na in plant tissues, especially in shoots; however, an increase in P and K was observed [15,17]. Our results indicate the potential impact of *Stutzerimonas stutzeri* PV248835, along with AMF, to enhance nutrient uptake and to reduce Na uptake under salinity stress.

### 4.6. AMF and STB 89 Reduce Proline Content and Activate Antioxidant Defense System in Maize Plants

Plants employ amino acids such as proline to maintain cellular osmotic and antioxidant balance during abiotic stress. In addition to being an osmoprotectant, proline can operate as a stress signal; hence, plants may acquire more proline due to a biotic stress or less proline as a result of decreased stress [43]. Our results showed a decrease in proline accumulation due to bioinoculants, and this decrease was more pronounced in the combined application (Figure 5A). The accumulation of low proline in the treated plants indicated that these plants were less exposed to salinity and did not require excessive proline accumulation to cope with salinity stress. Similarly, Echeverria et al. ref. [73] shows that AMF significantly decreased proline accumulation in lotus plants under salinity stress. In contrast, Elhindi et al. [74] observed an increase in proline accumulation in sweet basil plants under salinity stress. While Borde et al. [75] observed a decrease in proline accumulation in the shoots of the bajra plant due to AMF inoculation, proline accumulation in the roots was higher in mycorrhizal plants at all levels of salinity stress. Hence, these patterns may depend on several factors such as plant species, plant organs, and salinity level. On the other hand, we observed a significant increase in the activities of antioxidant enzymes (PPO, POX, and CAT) due to bioinoculants application (Figure 5B–D). Elevated enzyme activity indicates that maize plants engage their defensive systems of antioxidant enzymes to scavenge those producing ROS during salt stress. These results are consistent with those obtained by [2,76,77] in maize plants. These results indicate that the elevated enzyme activities resulting from bioinoculants application can protect maize plants from membrane damage caused by salinity stress.

## 5. Conclusions

Pressure on freshwater resources due to population growth is a major problem, resulting in a scarcity of water available for irrigation, which may necessitate the use of saline water for irrigation, which is problematic owing to the existence of plants sensitive to salinity, such as maize. Therefore, in this study, we isolated ninety-seven salt-tolerant bacterial isolates that may aid maize plants tolerate salinity stress. These bacteria could grow under different concentrations of NaCl, and nine isolates of them could produce growth-promoting substances effectively under salinity stress, in vitro. The most potent isolate (STB 89) was identified as *Stutzerimonas stutzeri* PV248835, and it was tested along with AMF on its ability to mitigate salinity stress in maize plants in a pot experiment. Our results showed that the inoculation of *Stutzerimonas stutzeri* PV248835 with AMF improved growth parameters, photosynthetic-related pigments, NPK content, and antioxidant enzymes while reducing proline accumulation, Na uptake, and the Na^+^/K^+^ ratio in maize plant tissues. In addition, bio-inoculation improved bacterial counts and mycorrhizal colonization under salinity stress. Taken together, inoculation of *Stutzerimonas stutzeri* PV248835 could support salinity stress mitigating the impact of AMF, and a cost-effective and easy-applicable technique could help farmers to mitigate salinity stress on maize plants. Future research should focus on signaling and molecular approaches to plant–microbe interactions, as well as expanding field experiments to study maize productivity under the combined effect of bio-inoculation and salinity.

## Figures and Tables

**Table 1:** Some chemical properties of the collected soil samples.

Soil Sample No.	Location	Soil Type	EC Values (dSm^−1^)	pH Values	Isolates Number	Isolates Key
S1	Klabsho	Sandy	10.25	8.59	12	STB: 14,27,32,77,78,79,80, 81,82, 83, 84,85, 87
S2	Klabsho	Sandy	2.63	8.55	15	STB: 24,25,26,28,29,30,31,33, 34,35,36,37,38,39,40
S3	Klabsho	Sandy	3.38	8.52	3	STB: 1,2,4
S4	North Delta	Clay	2.38	8.34	3	STB: 74,75,76
S5	Bahariya Oasis	Sandy	9.90	8.11	1	STB: 87
S6	Bahariya Oasis	Sandy	10.71	8.24	5	STB: 86,88,89,90,95
S7	Bahariya Oasis	Sandy	7.19	7.98	3	STB: 91,92,96
S8	Bahariya Oasis	Sandy	9.35	8.04	3	STB: 93,94,97
S9	El-Senbellawein	Clay	7.36	8.79	21	STB: 17,41,42,43,44,58,59, 60,61,62,63,64,65,66, 67,68,69,70,71,72,73
S10	Gamasa	Sandy	2.11	7.98	31	STB: 3,5,6,7,8,9,10,11,12, 13,15,16,18,19,20,21, 22,23,45,46,47,48,49, 50,51,52,53,54,55,56,57

**Table 2:** Physicochemical and biological properties of the experimental soil.

Property	Value
**Particle size distribution (%)**	
Coarse Sand	2.24
Fine Sand	23.51
Silt	42.00
Clay	32.26
Soil texture	Clay loam
**Physical and chemical analysis**	
OC %	0.42
OM %	0.73
pH	8.05
EC	1.64
**Cations (meq L^−1^)**	
Ca++	6.11
Mg++	2.97
Na+	9.24
K+	0.43
**Anions (meq L^−1^)**	
CO_3_^−2^	0.00
HCO_3_^−^	1.30
Cl^−^	5.79
SO_4_^−2^	11.65
**Available nutrients (mg kg^−1^)**	
N	67.56
P	19.66
K	343
**Bacterial count (Log (cfu g^−1^ dry soil))**	
TBC	6.238
PSBC	5.113
KRBC	4.369

OC Organic carbon; OM Organic Matter; pH (1:2.5); EC (electrical conductivity dSm^−1^); TBC Total bacterial count; PSBC phosphate-solubilizing bacterial count; KRBC potassium-releasing bacterial count.

**Table 3:** Counts of bacteria in the rhizosphere of maize and levels of AMF colonization (%) in the roots of maize plants grown under either control or salinity stress conditions.

Treatments		Count of Bacteria Log (cfu g^−1^ Dry Soil)			Levels of AMF Colonization (%)		
		TCB	PSBC	KRBC	F	M	A
**Control**	CK	7.876 ± 0.027e	5.825 ± 0.016ef	5.106 ± 0.005e	–	–	–
	AMF	7.972 ± 0.016d	5.974 ± 0.002c	5.154 ± 0.013d	90.91 ± 0.36ab	67.92 ± 1.87b	57.73 ± 0.78b
	STB 89	8.128 ± 0.043c	6.108 ± 0.048b	5.218 ± 0.006c	–	–	–
	Combination	8.267 ± 0.021a	6.259 ± 0.012a	5.288 ± 0.011a	91.67 ± 1.64a	72.27 ± 0.54a	63.37 ± 1.40a
50 mM NaCl	CK	7.535 ± 0.026h	5.771 ± 0.048f	5.037 ± 0.033g	–	–	–
	AMF	7.800 ± 0.038f	5.876 ± 0.012de	5.091 ± 0.004ef	81.82 ± 1.19c	61.00 ± 1.11d	40.95 ± 0.85d
	STB 89	7.980 ± 0.009d	5.936 ± 0.007cd	5.200 ± 0.006c	–	–	–
	Combination	8.196 ± 0.008b	6.110 ± 0.051b	5.247 ± 0.007b	90.00 ± 1.06b	62.50 ± 0.56c	42.80 ± 0.53c
100 mM NaCl	CK	7.314 ± 0.023i	5.624 ± 0.056g	5.027 ± 0.009g	–	–	–
	AMF	7.715 ± 0.021g	5.795 ± 0.043f	5.074 ± 0.008f	60.00 ± 0.45e	31.82 ± 1.33f	24.55 ± 0.63f
	STB 89	7.895 ± 0.020e	5.902 ± 0.016d	5.164 ± 0.015d	–	–	–
	Combination	8.098 ± 0.008c	5.965 ± 0.014c	5.221 ± 0.016c	72.73 ± 2.18d	35.50 ± 0.61e	27.82 ± 0.88e
Salinity		***	***	***	***	***	***
Bio-inoculation		***	***	***	***	***	***
Salinity × Bio-inoculation		***	**	ns	***	***	***

Data are means ± standard deviation; different letters within the same column indicate significance; ns, not significant; and *, **, *** denote significant differences between factors at *p* ≤ 0.05, 0.01, and 0.001, respectively. Ck, Check; AMF, Arbuscular mycorrhizal fungi; STB 89, Salinity tolerant bacteria “*Stutzerimonas stutzeri* PV248835”; TBC, Total bacterial count; PSBC, phosphate-solubilizing bacterial count; KRBC, potassium-releasing bacterial count; F%, the frequency of root colonization; M%, the intensity of cortical colonization; A%, arbuscular frequency in roots.

**Table 4:** Morphological response of maize plants grown under either control or salinity stress conditions.

Treatments		Shoot Length (cm)	Root Length (cm)	SDW (g Plant ^−1^)	RDW (g Plant ^−1^)	Leaf Area (cm^2^)
**Control**	CK	98.00 ± 5.98bc	16.97 ± 2.8ef	11.81 ± 0.68e	1.51 ± 0.46de	237.99 ± 18.5fg
	AMF	120.33 ± 4.04a	21.53 ± 0.4b	19.02 ± 3.93b	4.43 ± 0.17b	320.95 ± 5.8bc
	STB 89	107.93 ± 3.27b	20.87 ± 0.35b	17.08 ± 0.96bc	3.05 ± 0.61c	297.57 ± 16.76c–e
	Combination	125.7 ± 2.48a	25.43 ± 1.1a	24.61 ± 0.81a	5.41 ± 0.07a	366.57 ± 20.36a
50 mM NaCl	CK	80.53 ± 1.82de	14.27 ± 1.1g	8.38 ± 1.32fg	0.90 ± 0.08ef	196.98 ± 10.47h
	AMF	99.6 ± 13.43bc	18.93 ± 0.23cd	14.69 ± 0.56d	2.68 ± 0.64c	307.94 ± 4.08b–d
	STB 89	89.83 ± 9.17cd	17.07 ± 0.12ef	10.85 ± 0.79e	1.61 ± 0.31d	267.99 ± 6.41ef
	Combination	105.97 ± 3.41b	21.63 ± 0.78b	15.26 ± 1.03cd	4.64 ± 0.48b	334.88 ± 3.37b
100 mM NaCl	CK	61.57 ± 4.67f	12.13 ± 0.12h	4.50 ± 0.36h	0.52 ± 0.01f	130.88 ± 9.93i
	AMF	73.00 ± 1.30e	18.17 ± 0.47de	6.49 ± 0.08gh	0.94 ± 0.04ef	211.26 ± 8.02gh
	STB 89	72.77 ± 0.65e	15.97 ± 1.05fg	4.85 ± 0.14h	0.70 ± 0.05f	182.27 ± 12.32h
	Combination	81.43 ± 5.02de	20.07 ± 0.6bc	9.69 ± 0.09ef	1.42 ± 0.41de	286.57 ± 48.9de
Salinity		***	***	***	***	***
Bio-inoculation		***	***	***	***	***
Salinity × Bio-inoculation		ns	ns	***	***	ns

Data are means ± standard deviation; different letters within the same column indicate significance; ns, not significant; and *, **, *** denote significant differences between factors at *p* ≤ 0.05, 0.01, and 0.001, respectively. Ck, Check; AMF, Arbuscular mycorrhizal fungi; STB 89, Salinity tolerant bacteria “*Stutzerimonas stutzeri* PV248835”; SDW, Shoot Dry Weight; RDW, Root Dry Weight.

## Data Availability

The data presented in this study are available upon request from the corresponding author.

## Supplementary Materials

The following supporting information can be downloaded at: https://www.mdpi.com/article/10.3390/microorganisms13061345/s1, Table S1(A–D): Effect of NaCl concentrations on the growth of bacterial isolates; Table S2(A–D): (increase/decrease) percent (%) of growth compared to the control; Figure S1: A map showing the locations from where soil samples were collected; Figure S2: Phylogenetic tree of *Stutzerimonas stutzeri* PV248835.

## References

[1] Negm A.M., Omran E.-S.E., Mahmoud M.A., Abdel-Fattah S., Negm A.M. (2019). Update, Conclusions, and Recommendations for Conventional Water Resources and Agriculture in Egypt. Conventional Water Resources and Agriculture in Egypt.

[2] El-Sawah A.M., Abdel-Fattah G.G., Holford P., Korany S.M., Alsherif E.A., AbdElgawad H., Ulhassan Z., Jośko I., Ali B., Sheteiwy M.S. (2023). Funneliformis constrictum modulates polyamine metabolism to enhance tolerance of *Zea mays* L. to salinity. Microbiol. Res..

[3] Sang H., Guo W., Gao Y., Jiao X., Pan X. (2020). Effects of Alternating Fresh and Saline Water Irrigation on Soil Salinity and Chlorophyll Fluorescence of Summer Maize. Water.

[4] Liu C., Jiang X., Yuan Z. (2024). Plant Responses and Adaptations to Salt Stress: A Review. Horticulturae.

[5] Boorboori M.R., Lackóová L. (2025). Arbuscular mycorrhizal fungi and salinity stress mitigation in plants. Front. Plant Sci..

[6] Muhammad M., Waheed A., Wahab A., Majeed M., Nazim M., Liu Y.-H., Li L., Li W.-J. (2024). Soil salinity and drought tolerance: An evaluation of plant growth, productivity, microbial diversity, and amelioration strategies. Plant Stress.

[7] Evelin H., Devi T.S., Gupta S., Kapoor R. (2019). Mitigation of Salinity Stress in Plants by Arbuscular Mycorrhizal Symbiosis: Current Understanding and New Challenges. Front. Plant Sci..

[8] Balasubramaniam T., Shen G., Esmaeili N., Zhang H. (2023). Plants’ Response Mechanisms to Salinity Stress. Plants.

[9] Atta K., Mondal S., Gorai S., Singh A.P., Kumari A., Ghosh T., Roy A., Hembram S., Gaikwad D.J., Mondal S. (2023). Impacts of salinity stress on crop plants: Improving salt tolerance through genetic and molecular dissection. Front. Plant Sci..

[10] Zhang Z., Zhou Z., Feng S., Guo P., Wang Y., Hao B., Guo W., Li F.Y. (2024). Synergistic effects of AMF and PGPR on improving saline-alkaline tolerance of *Leymus chinensis* by strengthening the link between rhizosphere metabolites and microbiomes. Environ. Technol. Innov..

[11] Wen Y., Wu R., Qi D., Xu T., Chang W., Li K., Fang X., Song F. (2024). The effect of AMF combined with biochar on plant growth and soil quality under saline-alkali stress: Insights from microbial community analysis. Ecotoxicol. Environ. Saf..

[12] Khalloufi M., Martínez-Andújar C., Karray-Bouraouib N., Pérez-Alfocea F., Albacete A. (2024). The crosstalk interaction of ethylene, gibberellins, and arbuscular mycorrhiza improves growth in salinized tomato plants by modulating the hormonal balance. J. Plant Physiol..

[13] Huang P., Huang S., Ma Y., Danish S., Hareem M., Syed A., Elgorban A.M., Eswaramoorthy R., Wong L.S. (2024). Alleviation of salinity stress by EDTA chelated-biochar and arbuscular mycorrhizal fungi on maize via modulation of antioxidants activity and biochemical attributes. BMC Plant Biol..

[14] Fan L., Zhang P., Cao F., Liu X., Ji M., Xie M. (2024). Effects of AMF on Maize Yield and Soil Microbial Community in Sandy and Saline Soils. Plants.

[15] de Carvalho Neta S.J., Araújo V.L.V.P., Fracetto F.J.C., da Silva C.C.G., de Souza E.R., Silva W.R., Lumini E., Fracetto G.G.M. (2024). Growth-promoting bacteria and arbuscular mycorrhizal fungus enhance maize tolerance to saline stress. Microbiol. Res..

[16] Wang H., An T., Huang D., Liu R., Xu B., Zhang S., Deng X., Siddique K.H.M., Chen Y. (2021). Arbuscular mycorrhizal symbioses alleviating salt stress in maize is associated with a decline in root-to-leaf gradient of Na^+^/K^+^ ratio. BMC Plant Biol..

[17] Chandra P., Singh A., Prajapat K., Rai A.K., Yadav R.K. (2022). Native arbuscular mycorrhizal fungi improve growth, biomass yield, and phosphorus nutrition of sorghum in saline and sodic soils of the semi–arid region. Environ. Exp. Bot..

[18] Gao C., El-Sawah A.M., Ali D.F.I., Alhaj Hamoud Y., Shaghaleh H., Sheteiwy M.S. (2020). The Integration of Bio and Organic Fertilizers Improve Plant Growth, Grain Yield, Quality and Metabolism of Hybrid Maize (*Zea mays* L.). Agronomy.

[19] Xu W., Liu Q., Wang B., Zhang N., Qiu R., Yuan Y., Yang M., Wang F., Mei L., Cui G. (2024). Arbuscular mycorrhizal fungi communities and promoting the growth of alfalfa in saline ecosystems of northern China. Front. Plant Sci..

[20] Malik J.A., Alqarawi A.A., Alotaibi F., Habib M.M., Sorrori S.N., Almutairi M.B.R., Dar B.A. (2024). Alleviation of NaCl Stress on Growth and Biochemical Traits of *Cenchrus ciliaris* L. via Arbuscular Mycorrhizal Fungi Symbiosis. Life.

[21] Kakabouki I., Stavropoulos P., Roussis I., Mavroeidis A., Bilalis D. (2023). Contribution of Arbuscular Mycorrhizal Fungi (AMF) in Improving the Growth and Yield Performances of Flax (*Linum usitatissimum* L.) to Salinity Stress. Agronomy.

[22] Qin W., Yan H., Zou B., Guo R., Ci D., Tang Z., Zou X., Zhang X., Yu X., Wang Y. (2021). Arbuscular mycorrhizal fungi alleviate salinity stress in peanut: Evidence from pot-grown and field experiments. Food Energy Secur..

[23] Ghosh S., Bhowmik S., Dutta S.S., Parihar M., Rakshit A., Adholeya A., Chen Y. (2024). Challenges in Application of Arbuscular Mycorrhizal Inocula in Conventional Agriculture. Arbuscular Mycorrhizal Fungi in Sustainable Agriculture: Inoculum Production and Application.

[24] Szymańska S., Lis M.I., Piernik A., Hrynkiewicz K. (2022). *Pseudomonas stutzeri* and *Kushneria marisflavi* Alleviate Salinity Stress-Associated Damages in Barley, Lettuce, and Sunflower. Front. Microbiol..

[25] Lami M.J., Adler C., Caram-Di Santo M.C., Zenoff A.M., de Cristóbal R.E., Espinosa-Urgel M., Vincent P.A. (2020). *Pseudomonas stutzeri* MJL19, a rhizosphere-colonizing bacterium that promotes plant growth under saline stress. J. Appl. Microbiol..

[26] Szymańska S., Dąbrowska G.B., Tyburski J., Niedojadło K., Piernik A., Hrynkiewicz K. (2019). Boosting the Brassica napus L. tolerance to salinity by the halotolerant strain *Pseudomonas stutzeri* ISE12. Environ. Exp. Bot..

[27] Ke X., Feng S., Wang J., Lu W., Zhang W., Chen M., Lin M. (2019). Effect of inoculation with nitrogen-fixing bacterium *Pseudomonas stutzeri* A1501 on maize plant growth and the microbiome indigenous to the rhizosphere. Syst. Appl. Microbiol..

[28] Bacilio M., Moreno M., Bashan Y. (2016). Mitigation of negative effects of progressive soil salinity gradients by application of humic acids and inoculation with *Pseudomonas stutzeri* in a salt-tolerant and a salt-susceptible pepper. Appl. Soil Ecol..

[29] Egamberdieva D., Wirth S., Bellingrath-Kimura S.D., Mishra J., Arora N.K. (2019). Salt-Tolerant Plant Growth Promoting Rhizobacteria for Enhancing Crop Productivity of Saline Soils. Front. Microbiol..

[30] Patel T., Saraf M. (2017). Biosynthesis of phytohormones from novel rhizobacterial isolates and their in vitro plant growth-promoting efficacy. J. Plant Interact..

[31] Sadeghi A., Karimi E., Dahaji P.A., Javid M.G., Dalvand Y., Askari H. (2012). Plant growth promoting activity of an auxin and siderophore producing isolate of Streptomyces under saline soil conditions. World J. Microbiol. Biotechnol..

[32] Tiwari G., Duraivadivel P., Sharma S., Hariprasad P. (2018). 1-Aminocyclopropane-1-carboxylic acid deaminase producing beneficial rhizobacteria ameliorate the biomass characters of Panicum maximum Jacq. by mitigating drought and salt stress. Sci. Rep..

[33] Qurashi A., Sabri A. (2012). Bacterial exopolysaccharide and biofilm formation stimulate chickpea growth and soil aggregation under salt stress. Braz. J. Microbiol..

[34] Saum S.H., Müller V. (2007). Salinity-Dependent Switching of Osmolyte Strategies in a Moderately Halophilic Bacterium: Glutamate Induces Proline Biosynthesis in *Halobacillus halophilus*. J. Bacteriol..

[35] Bharti N., Pandey S.S., Barnawal D., Patel V.K., Kalra A. (2016). Plant growth promoting rhizobacteria *Dietzia natronolimnaea* modulates the expression of stress responsive genes providing protection of wheat from salinity stress. Sci. Rep..

[36] Kohler J., Hernández J.A., Caravaca F., Roldán A. (2009). Induction of antioxidant enzymes is involved in the greater effectiveness of a PGPR versus AM fungi with respect to increasing the tolerance of lettuce to severe salt stress. Environ. Exp. Bot..

[37] Patel D., Saraf M. (2013). Influence of soil ameliorants and microflora on induction of antioxidant enzymes and growth promotion of *Jatropha curcas* L. under saline condition. Eur. J. Soil Biol..

[38] Tu Q., Tang S., Huang S. (2025). Mitigation of salinity stress via improving growth, chlorophyll contents and antioxidants defense in sunflower with *Bacillus pumilis* and biochar. Sci. Rep..

[39] Sridhar D., Alheswairini S.S., Barasarathi J., Enshasy H.A.E., Lalitha S., Mir S.H., Nithyapriya S., Sayyed R. (2025). Halophilic rhizobacteria promote growth, physiology and salinity tolerance in Sesamum indicum L. grown under salt stress. Front. Microbiol..

[40] Chen Z., Zhang P., Wang B., Li H., Li S., Zhang H., Haider F.U., Li X. (2025). Harnessing the role of rhizo-bacteria to mitigate salinity stress in rice (*Orzya sativa*); focus on antioxidant defense system, photosynthesis response, and rhizosphere microbial diversity. Rhizosphere.

[41] Farooq M., Hussain M., Wakeel A., Siddique K.H.M. (2015). Salt stress in maize: Effects, resistance mechanisms, and management. A review. Agron. Sustain. Dev..

[42] Jackson M.L. (2005). Soil Chemical Analysis: Advanced Course: A Manual of Methods Useful for Instruction and Research in Soil Chemistry, Physical Chemistry of Soils, Soil Fertility, and Soil Genesis.

[43] Nader A.A., Hauka F.I.A., Afify A.H., El-Sawah A.M. (2024). Drought-Tolerant Bacteria and Arbuscular Mycorrhizal Fungi Mitigate the Detrimental Effects of Drought Stress Induced by Withholding Irrigation at Critical Growth Stages of Soybean (*Glycine max*, L.). Microorganisms.

[44] Abdelsattar M., El-Sawah A.M., El-Kady S., Hauka F.I.A. (2022). Evaluation of Plant Growth Promoting of Salt-tolerant Rhizobacteria Isolated from Egyptian Saline Soils. J. Agric. Chem. Biotechnol..

[45] Ahmad F., Ahmad I., KHAN M.S. (2005). Indole acetic acid production by the indigenous isolates of Azotobacter and fluorescent Pseudomonas in the presence and absence of tryptophan. Turk. J. Biol..

[46] Abou-Aly H.E., Youssef A.M., El-Meihy R.M., Tawfik T.A., El-Akshar E.A. (2019). Evaluation of heavy metals tolerant bacterial strains as antioxidant agents and plant growth promoters. Biocatal. Agric. Biotechnol..

[47] Pikovskaya R. (1948). Mobilization of phosphorus in soil in connection with vital activity of some microbial species. Mikrobiologiya.

[48] Boltz D.F., Mellon M.G. (1948). Spectrophotometric Determination of Phosphorus as Molybdiphosphoric Acid. Anal. Chem..

[49] Hemalatha N., Raja N., Jayachitra A., Rajalakshmi A., Valarmathi N. (2013). Isolation and characterization of phosphate solubilizing bacteria and analyzing their effect on Capsicum annum L.. Inter. J. Biol. Pharm. Res.

[50] Sharath S., Triveni S., Nagaraju Y., Latha P.C., Vidyasagar B. (2021). The Role of Phyllosphere Bacteria in Improving Cotton Growth and Yield Under Drought Conditions. Front. Agron..

[51] Bates L.S., Waldren R.P., Teare I.D. (1973). Rapid determination of free proline for water-stress studies. Plant Soil.

[52] Skerman V.B. (1967). A Guide to the Identification of the Genera of Bacteria.

[53] Setiawati T.C., Mutmainnah L. (2016). Solubilization of potassium containing mineral by microorganisms from sugarcane rhizosphere. Agric. Agric. Sci. Procedia.

[54] Phillips J., Hayman D. (1970). Improved procedures for clearing roots and staining parasitic and vesicular-arbuscular mycorrhizal fungi for rapid assessment of infection. Trans. Br. Mycol. Soc..

[55] Trouvelot A., Kough J.L., Gianinazzi-Pearson V. (1986). Measure du taux de mycorrhization d’un systeme radiculaire. Recherche de methods d’estimation ayant une signification fonctionnelle. Physiological and Genetical Aspects of Mycorrhizae.

[56] Amanullah H., Marwat K., Shah P., Maula N., Arifullah S. (2009). Nitrogen levels and its time of application influence leaf area, height and biomass of maize planted at low and high density. Pak. J. Bot.

[57] Lichtenthaler H.K., Wellburn A.R. (1983). Determinations of total carotenoids and chlorophylls a and b of leaf extracts in different solvents. Biochem. Soc. Trans..

[58] Snell F.D., Snell C.T. (1961). Colorimetric Methods of Analysis.

[59] Chapman H.D., Pratt P.F. (1962). Methods of analysis for soils, plants and waters. Soil Sci..

[60] Malik C.P., Singh M.B. (1980). Plant Enzymology and Histo Enzymology.

[61] Hammerschmidt R., Nuckles E.M., Kuć J. (1982). Association of enhanced peroxidase activity with induced systemic resistance of cucumber to *Colletotrichum lagenarium*. Physiol. Plant Pathol..

[62] Aebi H.E., Bergmeyer H.U. (1983). Catalase. Methods of Enzymatic Analysis.

[63] Alonazi M.A., Alwathnani H.A., AL-Barakah F.N.I., Alotaibi F. (2025). Native Plant Growth-Promoting Rhizobacteria Containing ACC Deaminase Promote Plant Growth and Alleviate Salinity and Heat Stress in Maize (*Zea mays* L.) Plants in Saudi Arabia. Plants.

[64] Afify A., Ashour A. (2018). Use of Cyanobacteria for Controlling Flax Seedling Blight. J. Agric. Chem. Biotechnol..

[65] Mahmoud A.M., Reyad A.M., Khalaf M.H., Sheteiwy M.S., Dawood M.F.A., El-Sawah A.M., Shaban Ahmed E., Malik A., Al-Qahtani W.H., Abdel-Maksoud M.A. (2024). Investigating the Endophyte *Actinomycetota* sp. JW0824 Strain as a Potential Bioinoculant to Enhance the Yield, Nutritive Value, and Chemical Composition of Different Cultivars of Anise (*Pimpinella anisum* L.) Seeds. Biology.

[66] El-Amriti F.A., Ouf S.A., Abu-Elghait M., Desouky S.E., Mohamed M.S.M. (2024). Alleviation of salt stress on *Zea mays* L. plant by PGPR isolates as an effective sustainable strategy. Biocatal. Agric. Biotechnol..

[67] Mahmoud A.M., Sheteiwy M.S., El-Keblawy A., Ulhassan Z., Khalaf M.H., Mohamed H.S., Okla M.K., AlGarawi A.M., El-Sawah A.M., Ahmed E.S. (2025). The potential biofortification role of *Actinopolyspora* sp. JTT-01 in enhancing the yield and tissue chemical composition of caraway plants. BMC Plant Biol..

[68] Kumar A., Singh S., Mukherjee A., Rastogi R.P., Verma J.P. (2021). Salt-tolerant plant growth-promoting *Bacillus pumilus* strain JPVS11 to enhance plant growth attributes of rice and improve soil health under salinity stress. Microbiol. Res..

[69] Shahid M., Altaf M., Ali S., Tyagi A. (2024). Isolation and assessment of the beneficial effect of exopolysaccharide-producing PGPR in *Triticum aestivum* (L.) plants grown under NaCl and Cd-stressed conditions. Plant Physiol. Biochem..

[70] Juniper S., Abbott L.K. (2006). Soil salinity delays germination and limits growth of hyphae from propagules of arbuscular mycorrhizal fungi. Mycorrhiza.

[71] Pan J., Huang C., Peng F., Wang T., Liao J., Ma S., You Q., Xue X. (2022). Synergistic combination of arbuscular mycorrhizal fungi and plant growth-promoting rhizobacteria modulates morpho-physiological characteristics and soil structure in *Nitraria tangutorum* bobr. Under saline soil conditions. Res. Cold Arid Reg..

[72] Chandrasekaran M., Boopathi T., Manivannan P. (2021). Comprehensive Assessment of Ameliorative Effects of AMF in Alleviating Abiotic Stress in Tomato Plants. J. Fungi.

[73] Echeverria M., Sannazzaro A.I., Ruiz O.A., Menéndez A.B. (2013). Modulatory effects of *Mesorhizobium tianshanense* and *Glomus intraradices* on plant proline and polyamine levels during early plant response of Lotus tenuis to salinity. Plant Soil.

[74] Elhindi K.M., El-Din A.S., Elgorban A.M. (2017). The impact of arbuscular mycorrhizal fungi in mitigating salt-induced adverse effects in sweet basil (*Ocimum basilicum* L.). Saudi J. Biol. Sci..

[75] Borde M., Dudhane M., Jite P. (2011). Growth photosynthetic activity and antioxidant responses of mycorrhizal and non-mycorrhizal bajra (*Pennisetum glaucum*) crop under salinity stress condition. Crop Prot..

[76] Ouhaddou R., Meddich A., Ikan C., Lahlali R., Ait Barka E., Hajirezaei M.-R., Duponnois R., Baslam M. (2023). Enhancing Maize Productivity and Soil Health under Salt Stress through Physiological Adaptation and Metabolic Regulation Using Indigenous Biostimulants. Plants.

[77] Shabaan M., Asghar H.N., Zahir Z.A., Zhang X., Sardar M.F., Li H. (2022). Salt-Tolerant PGPR Confer Salt Tolerance to Maize Through Enhanced Soil Biological Health, Enzymatic Activities, Nutrient Uptake and Antioxidant Defense. Front. Microbiol..

