# Supplementary material for: Salt-Tolerant Bacteria Support Salinity Stress Mitigating Impact of Arbuscular Mycorrhizal Fungi in Maize (Zea mays L.)
Source: Microorganisms. 2025 Jun 10;13(6):1345. doi: 10.3390/microorganisms13061345 (PMC12196076; doi:10.3390/microorganisms13061345)
Supplement: Supplementary file 1 [file microorganisms-13-01345-s001.zip › microorganisms-3645727-supplementary.pdf]

Salt-Tolerant Bacteria Support Salinity Stress Mitigating Impact of Arbuscular Mycorrhizal Fungi in Maize (*Zea mays*, L.)

Randa M. Zaki [1], Aida H. Afify [1], Eman H. Ashour [1], Ahmed M. El-Sawah [1*]

[1] Department of Agricultural Microbiology, Faculty of Agriculture, Mansoura University, Mansoura 35516, Egypt

*Correspondence: ahmedelsawah89@mans.edu.eg (A.M.E-S.)

Table S1A. Effect of NaCl concentrations on the growth of bacterial isolates.

Isolate No.	Growth of bacterial isolates expressed as OD (600 nm) at various salinity concentrations.					
	0% NaCl	3% NaCl	6% NaCl	9% NaCl	12% NaCl	15% NaCl
**STB 1**	0.645±0.027m-z	0.668±0.004l-z	0.313±0.083r-D	0.211±0.060l-A	0.076±0.060j-u	0.045±0.030f-j
**STB 2**	0.604±0.037m-A	0.486±0.029r-I	0.366±0.018o-D	0.244±0.076j-A	0.067±0.012j-u	0.043±0.006g-j
**STB 3**	0.341±0.030r-A	0.185±0.024E-J	0.144±0.026z-D	0.126±0.048t-A	0.043±0.016k-u	0.023±0.011g-j
**STB 4**	0.362±0.157q-A	0.329±0.067v-J	0.223±0.032u-D	0.147±0.110q-A	0.068±0.053j-u	0.042±0.017g-j
**STB 5**	0.708±0.163m-x	0.337±0.266u-J	0.196±0.169w-D	0.101±0.058v-A	0.071±0.002j-u	0.067±0.004e-j
**STB 6**	0.225±0.084x-A	0.163±0.131F-J	0.041±0.009D	0.021±0.002z-A	0.031±0.017n-u	0.025±0.010g-j
**STB 7**	0.396±0.080p-A	0.606±0.320m-D	0.115±0.072B-D	0.144±0.008q-A	0.009±0.007s-u	0.000±0.000j
**STB 8**	0.158±0.097z-A	0.251±0.032A-J	0.164±0.023y-D	0.126±0.024t-A	0.067±0.019j-u	0.043±0.030g-j
**STB 9**	0.556±0.080m-A	0.306±0.113w-J	0.124±0.031A-D	0.163±0.114p-A	0.102±0.017j-u	0.095±0.012d-j
**STB 10**	0.393±0.058p-A	0.607±0.284m-D	0.208±0.066v-D	0.173±0.031o-A	0.090±0.061j-u	0.024±0.003g-j
**STB 11**	0.426±0.302o-A	0.146±0.041G-J	0.305±0.4106r-D	0.178±0.173n-A	0.050±0.015k-u	0.058±0.016f-j
**STB 12**	0.660±0.403m-y	0.214±0.015D-J	0.231±0.043u-D	0.306±0.063g-w	0.041±0.024l-u	0.030±0.020g-j
**STB 13**	0.471±0.423n-A	0.334±0.130u-J	0.262±0.102t-D	0.071±0.069w-A	0.035±0.008l-u	0.012±0.015h-j
**STB 14**	0.427±0.071o-A	0.225±0.124C-J	0.093±0.026C-D	0.127±0.019s-A	0.019±0.014q-u	0.012±0.011h-j
**STB 15**	0.74±0.0810k-w	0.476±0.159r-I	0.274±0.023s-D	0.228±0.066j-A	0.144±0.090g-s	0.032±0.008g-j
**STB 16**	0.531±0.152n-A	0.583±0.057n-E	0.422±0.156m-C	0.067±0.017w-A	0.056±0.044j-u	0.031±0.019g-j
**STB 17**	0.734±0.396l-w	0.310±0.099w-J	0.218±0.030u-D	0.167±0.014p-A	0.072±0.036j-u	0.036±0.016g-j
**STB 18**	0.425±0.010o-A	0.377±0.027t-J	0.290±0.105r-D	0.089±0.007v-A	0.037±0.002l-u	0.043±0.014g-j
**STB 19**	0.504±0.117n-A	0.342±0.139u-J	0.150±0.073z-D	0.024±0.015z-A	0.030±0.012n-u	0.039±0.006g-j
**STB 20**	0.486±0.112n-A	0.390±0.012t-J	0.178±0.085x-D	0.126±0.003t-A	0.079±0.063j-u	0.030±0.009g-j
**STB 21**	0.371±0.139q-A	0.142±0.083G-J	0.038±0.021D	0.025±0.012z-A	0.022±0.007p-u	0.011±0.002h-j
**STB 22**	0.294±0.115v-A	0.461±0.095r-J	0.218±0.090u-D	0.060±0.047x-A	0.045±0.008k-u	0.046±0.011f-j
**STB 23**	0.148±0.037A	0.415±0.068s-J	0.160±0.063y-D	0.041±0.012y-A	0.019±0.011q-u	0.015±0.013h-j
**STB 24**	0.868±0.034f-p	1.887±0.215a	0.906±0.317d-i	0.473±0.070d-j	0.186±0.017f-j	0.150±0.013c-h
**STB 25**	1.842±0.166ab	1.777±0.113ab	1.222±0.213a-d	0.577±0.149b-f	0.168±0.067g-m	0.096±0.024d-j

Data included are means ± SD; different letters within the same column indicate significant differences between means.

Table S1B. Effect of NaCl concentrations on the growth of bacterial isolates.

Isolate No.	Growth of bacterial isolates expressed as OD (600 nm) at various salinity concentrations.					
	**0% NaCl**	**3% NaCl**	**6% NaCl**	**9% NaCl**	**12% NaCl**	**15% NaCl**
**STB 26**	0.629±0.141m-A	0.386±0.006t-J	0.309±0.139r-D	0.152±0.022q-A	0.110±0.0101j-u	0.067±0.009e-j
**STB 27**	1.223±0.335c-l	1.298±0.214c-h	0.668±0.239h-q	0.220±0.117k-A	0.091±0.010j-u	0.073±0.029e-j
**STB 28**	0.289±0.083v-A	0.179±0.012E-J	0.146±0.021z-D	0.081±0.006w-A	0.051±0.008j-u	0.026±0.014g-j
**STB 29**	0.804±0.027h-t	0.740±0.019k-u	0.711±0.069f-o	0.526±0.074c-g	0.437±0.085cd	0.147±0.046c-i
**STB 30**	0.563±0.016m-A	0.456±0.088r-J	0.462±0.021l-B	0.366±0.054e-t	0.261±0.022e-g	0.162±0.074c-g
**STB 31**	0.652±0.044m-y	0.574±0.020n-E	0.520±0.021k-x	0.462±0.068d-k	0.179±0.068f-k	0.106±0.015c-j
**STB 32**	0.768±0.303i-v	1.194±0.112d-i	0.919±0.152c-h	0.619±0.244b-d	0.098±0.027j-u	0.068±0.010e-j
**STB 33**	1.272±0.298c-h	1.614±0.270a-c	1.483±0.143a	0.788±0.213b	0.596±0.054b	0.187±0.059c-f
**STB 34**	0.551±0.043m-A	0.286±0.103y-J	0.173±0.009x-D	0.150±0.026q-A	0.148±0.023g-r	0.078±0.037e-j
**STB 35**	0.508±0.112n-A	0.296±0.034x-J	0.172±0.098x-D	0.158±0.039q-A	0.124±0.066h-u	0.081±0.017e-j
**STB 36**	0.685±0.106m-x	0.607±0.076m-D	0.445±0.056l-B	0.303±0.062g-x	0.164±0.046g-n	0.081±0.021e-j
**STB 37**	0.840±0.376g-q	0.954±0.223g-n	1.236±0.120a-d	0.727±0.154bc	0.040±0.028l-u	0.038±0.010g-j
**STB 38**	0.152±0.039A	0.149±0.051G-J	0.135±0.034A-D	0.120±0.066u-A	0.113±0.049j-u	0.062±0.041f-j
**STB 39**	0.761±0.172j-v	0.490±0.276q-I	0.162±0.030y-D	0.143±0.030r-A	0.060±0.018j-u	0.057±0.036f-j
**STB 40**	1.959±0.074a	1.654±0.163a-c	1.037±0.148c-g	0.499±0.144c-h	0.445±0.093cd	0.103±0.036c-j
**STB 41**	1.331±0.075c-g	1.287±0.156c-h	1.103±0.237b-e	0.794±0.178b	0.405±0.056cd	0.370±0.066b
**STB 42**	1.528±0.171a-c	1.360±0.043c-g	0.834±0.094e-k	0.602±0.083b-e	0.454±0.0506c	0.210±0.067c-e
**STB 43**	0.461±0.025o-A	0.272±0.192z-J	0.143±0.091z-D	0.142±0.035s-A	0.021±0.016p-u	0.010±0.010h-j
**STB 44**	0.779±0.0103i-v	0.192±0.043E-J	0.018±0.010D	0.008±0.002A	0.000±0.000u	0.000±0.000j
**STB 45**	0.356±0.030q-A	0.162±0.014F-J	0.129±0.030A-D	0.068±0.016w-A	0.023±0.008p-u	0.013±0.003h-j
**STB 46**	0.228±0.042x-A	0.128±0.017H-J	0.049±0.004D	0.040±0.003y-A	0.017±0.001q-u	0.009±0.004h-j
**STB 47**	0.263±0.012w-A	0.209±0.018D-J	0.178±0.012x-D	0.147±0.009q-A	0.041±0.007l-u	0.027±0.002g-j
**STB 48**	0.466±0.045o-A	0.626±0.080m-C	0.895±0.061d-j	0.081±0.008w-A	0.035±0.009l-u	0.016±0.009h-j
**STB 49**	0.375±0.013q-A	0.313±0.015v-J	0.285±0.008s-D	0.291±0.106g-x	0.037±0.005l-u	0.018±0.002h-j
**STB 50**	0.353±0.038q-A	0.708±0.120k-w	0.123±0.050A-D	0.105±0.028u-A	0.030±0.019n-u	0.017±0.002h-j

Data included are means ± SD; different letters within the same column indicate significant differences between means.

Table S1C. Effect of NaCl concentrations on the growth of bacterial isolates.

Isolate No.	Growth of bacterial isolates expressed as OD (600 nm) at various salinity concentrations.					
	**0% NaCl**	**3% NaCl**	**6% NaCl**	**9% NaCl**	**12% NaCl**	**15% NaCl**
**STB 51**	0.603±0.119m-A	0.458±0.029r-J	0.258±0.012t-D	0.144±0.044q-A	0.046±0.018k-u	0.018±0.015h-j
**STB 52**	0.569±0.014m-A	0.468±0.055r-J	0.250±0.035t-D	0.172±0.043o-A	0.044±0.014k-u	0.004±0.004ij
**STB 53**	0.829±0.173h-r	0.938±0.021h-p	0.778±0.038e-l	0.371±0.029e-s	0.056±0.016j-u	0.046±0.009f-j
**STB 54**	0.708±0.036m-x	0.943±0.013h-o	0.437±0.027l-C	0.162±0.013q-A	0.061±0.018j-u	0.031±0.005g-j
**STB 55**	1.256±0.030c-i	0.443±0.025r-J	0.328±0.017q-D	0.172±0.012o-A	0.059±0.003j-u	0.039±0.013g-j
**STB 56**	0.638±0.042m-A	0.296±0.017x-J	0.205±0.046v-D	0.163±0.033p-A	0.054±0.017j-u	0.019±0.003g-j
**STB 57**	0.714±0.096m-x	0.528±0.071q-I	0.421±0.019n-C	0.163±0.023p-A	0.083±0.017j-u	0.054±0.005f-j
**STB 58**	0.497±0.165n-A	0.226±0.017C-J	0.165±0.043y-D	0.042±0.011y-A	0.017±0.001r-u	0.007±0.007h-j
**STB 59**	0.337±0.092s-A	0.531±0.176p-H	0.437±0.066l-C	0.235±0.070j-A	0.109±0.047j-u	0.243±0.318bc
**STB 60**	0.393±0.042p-A	0.703±0.151k-x	0.566±0.2306i-u	0.333±0.159f-v	0.120±0.029h-u	0.085±0.010e-j
**STB 61**	0.566±0.143m-A	0.898±0.265h-q	0.164±0.076y-D	0.094±0.004v-A	0.028±0.015o-u	0.012±0.003h-j
**STB 62**	0.883±0.011f-p	0.963±0.031g-n	0.623±0.010h-s	0.154±0.026q-A	0.108±0.008j-u	0.064±0.005f-j
**STB 63**	0.413±0.084o-A	0.570±0.108n-F	0.741±0.081f-n	0.422±0.044d-n	0.080±0.038j-u	0.047±0.009f-j
**STB 64**	0.893±0.010f-o	1.425±0.062b-e	1.057±0.138b-f	0.249±0.042i-A	0.141±0.048g-t	0.026±0.039g-j
**STB 65**	0.314±0.066t-A	0.343±0.036u-J	0.223±0.072u-D	0.126±0.030t-A	0.037±0.012l-u	0.014±0.007h-j
**STB 66**	0.309±0.045u-A	0.120±0.073I-J	0.115±0.053B-D	0.046±0.023y-A	0.043±0.028k-u	0.005±0.003ij
**STB 67**	0.367±0.022q-A	0.432±0.024s-J	0.313±0.019r-D	0.271±0.018h-y	0.031±0.016n-u	0.005±0.003ij
**STB 68**	1.232±0.138c-j	0.537±0.019o-G	0.475±0.018l-A	0.388±0.066d-q	0.076±0.003j-u	0.052±0.016f-j
**STB 69**	0.868±0.032f-p	0.462±0.128r-J	0.342±0.070p-D	0.265±0.040h-z	0.059±0.049j-u	0.017±0.005h-j
**STB 70**	0.332±0.038s-A	0.269±0.046z-J	0.212±0.062v-D	0.204±0.040m-A	0.065±0.026j-u	0.054±0.019f-j
**STB 71**	0.551±0.024m-A	0.352±0.033u-J	0.220±0.009u-D	0.079±0.006w-A	0.031±0.002n-u	0.013±0.005h-j
**STB 72**	0.551±0.091m-A	0.246±0.026B-J	0.145±0.036z-D	0.087±0.008w-A	0.036±0.002l-u	0.018±0.005h-j
**STB 73**	0.363±0.007q-A	0.292±0.024y-J	0.166±0.038y-D	0.025±0.010z-A	0.007±0.006t-u	0.001±0.001j
**STB 74**	1.394±0.140b-e	0.803±0.1708i-s	0.637±0.030h-r	0.411±0.059d-o	0.340±0.012c-e	0.236±0.087b-d
**STB 75**	0.838±0.079h-q	0.681±0.110l-y	0.534±0.071k-w	0.490±0.073c-i	0.412±0.011cd	0.094±0.017d-j

Data included are means ± SD; different letters within the same column indicate significant differences between means.

Table S1D. Effect of NaCl concentrations on the growth of bacterial isolates.

Isolate No.	Growth of bacterial isolates expressed as OD (600 nm) at various salinity concentrations.					
	0% NaCl	3% NaCl	6% NaCl	9% NaCl	12% NaCl	15% NaCl
**STB 76**	1.033±0.167d-m	0.613±0.071m-D	0.533±0.109k-w	0.128±0.032s-A	0.067±0.023j-u	0.063±0.018f-j
**STB 77**	0.351±0.088q-A	0.285±0.006y-J	0.244±0.077u-D	0.240±0.017j-A	0.157±0.051g-p	0.086±0.030e-j
**STB 78**	1.229±0.017c-k	0.581±0.004n-E	0.505±0.003k-y	0.387±0.017d-r	0.171±0.025g-l	0.058±0.015f-j
**STB 79**	1.031±0.183d-m	0.765±0.110j-t	0.671±0.073h-q	0.460±0.078d-k	0.139±0.050g-t	0.112±0.018c-j
**STB 80**	1.435±0.047b-e	0.659±0.037l-A	0.540±0.030k-w	0.468±0.101d-j	0.314±0.027d-f	0.137±0.010c-j
**STB 81**	1.501±0.128a-d	0.638±0.108m-B	0.495±0.077k-z	0.365±0.044e-t	0.255±0.009e-h	0.070±0.025e-j
**STB 82**	0.960±0.077e-n	0.843±0.078i-r	0.537±0.039k-w	0.349±0.027f-u	0.115±0.015i-u	0.098±0.008d-j
**STB 83**	0.769±0.175i-v	0.720±0.040k-v	0.552±0.090j-v	0.424±0.028d-m	0.162±0.063g-o	0.079±0.004e-j
**STB 84**	0.746±0.019j-w	0.502±0.052q-I	0.433±0.024l-C	0.123±0.025t-A	0.058±0.009j-u	0.036±0.004g-j
**STB 85**	0.478±0.050n-A	0.393±0.003t-J	0.325±0.019q-D	0.245±0.012i-A	0.120±0.013h-u	0.086±0.016e-j
**STB 86**	0.872±0.050f-p	1.010±0.065f-m	0.474±0.000l-A	0.455±0.012d-l	0.265±0.068e-g	0.039±0.037g-j
**STB 87**	1.479±0.080a-d	1.170±0.180d-j	0.784±0.016e-l	0.407±0.073d-p	0.095±0.028j-u	0.052±0.013f-j
**STB 88**	1.631±0.006a-c	1.410±0.126b-f	1.404±0.076ab	1.210±0.091a	0.811±0.159a	0.620±0.058a
**STB 89**	1.474±0.212a-d	1.948±0.057a	1.264±0.018a-c	1.109±0.018a	0.873±0.097a	0.546±0.046a
**STB 90**	0.816±0.017h-s	1.472±0.099b-d	0.620±0.177h-s	0.565±0.020b-f	0.112±0.019j-u	0.061±0.007f-j
**STB 91**	1.529±0.089a-c	1.063±0.089e-l	0.773±0.177e-m	0.060±0.009x-A	0.057±0.013j-u	0.046±0.013f-j
**STB 92**	0.792±0.057h-u	0.424±0.019s-J	0.169±0.023x-D	0.104±0.004v-A	0.028±0.005o-u	0.013±0.007h-j
**STB 93**	0.171±0.041y-A	0.062±0.030J	0.031±0.005D	0.021±0.002z-A	0.015±0.002r-u	0.012±0.008h-j
**STB 94**	0.452±0.028o-A	0.181±0.066E-J	0.111±0.011B-D	0.032±0.018y-A	0.031±0.021n-u	0.016±0.014h-j
**STB 95**	1.029±0.062d-m	0.943±0.026h-o	0.599±0.034h-t	0.387±0.047d-r	0.251±0.010e-i	0.051±0.028f-j
**STB 96**	1.531±0.287a-c	1.109±0.229d-k	0.712±0.077f-o	0.174±0.018o-A	0.154±0.023g-q	0.106±0.020c-j
**STB 97**	1.335±0.052c-f	1.013±0.113f-m	0.694±0.043g-p	0.123±0.007t-A	0.033±0.005m-u	0.016±0.002h-j

Data included are means ± SD; different letters within the same column indicate significant differences between means.

Table S2A. (increase/decrease) percent (%) of growth compared to the control.

Isolate No.	3% NaCl	6% NaCl	9% NaCl	12% NaCl	15% NaCl
**STB 1**	+4%	-51%	-67%	-88%	-93%
**STB 2**	-20%	-39%	-60%	-89%	-93%
**STB 3**	-46%	-58%	-63%	-87%	-93%
**STB 4**	-9%	-38%	-59%	-81%	-88%
**STB 5**	-52%	-72%	-86%	-90%	-91%
**STB 6**	-28%	-82%	-91%	-86%	-89%
**STB 7**	+53%	-71%	-64%	-98%	-100%
**STB 8**	+9%	+4%	-20%	-58%	-73%
**STB 9**	-45%	-78%	-71%	-82%	-83%
**STB 10**	+54%	-47%	-56%	-77%	-94%
**STB 11**	-66%	-28%	-58%	-88%	-86%
**STB 12**	-68%	-65%	-54%	-94%	-95%
**STB 13**	-29%	-44%	-85%	-93%	-97%
**STB 14**	-47%	-78%	-70%	-96%	-97%
**STB 15**	-36%	-63%	-69%	-81%	-96%
**STB 16**	+10%	-21%	-87%	-89%	-94%
**STB 17**	-58%	-70%	-77%	-90%	-95%
**STB 18**	-11%	-32%	-79%	-91%	-90%
**STB 19**	-32%	-70%	-95%	-94%	-92%
**STB 20**	-20%	-63%	-74%	-84%	-94%
**STB 21**	-62%	-90%	-93%	-94%	-97%
**STB 22**	+57%	-26%	-80%	-85%	-84%
**STB 23**	+180%	+8%	-72%	-87%	-90%
**STB 24**	+117%	+4%	-46%	-79%	-83%
**STB 25**	-4%	-34%	-69%	-91%	-95%

Table S2B. (increase/decrease) percent (%) of growth compared to the control.

Isolate No.	3% NaCl	6% NaCl	9% NaCl	12% NaCl	15% NaCl
**STB 26**	-39%	-51%	-76%	-83%	-89%
**STB 27**	+6%	-45%	-82%	-93%	-94%
**STB 28**	-38%	-49%	-72%	-82%	-91%
**STB 29**	-8%	-12%	-35%	-46%	-82%
**STB 30**	-19%	-18%	-35%	-54%	-71%
**STB 31**	-12%	-20%	-29%	-73%	-84%
**STB 32**	+55%	+20%	-19%	-87%	-91%
**STB 33**	+27%	+17%	-38%	-53%	-85%
**STB 34**	-48%	-69%	-73%	-73%	-86%
**STB 35**	-42%	-66%	-69%	-76%	-84%
**STB 36**	-11%	-35%	-56%	-76%	-88%
**STB 37**	+14%	+47%	-13%	-95%	-95%
**STB 38**	-2%	-11%	-21%	-26%	-59%
**STB 39**	-36%	-79%	-81%	-92%	-93%
**STB 40**	-16%	-47%	-75%	-77%	-95%
**STB 41**	-3%	-17%	-40%	-70%	-72%
**STB 42**	-11%	-45%	-61%	-70%	-86%
**STB 43**	-41%	-69%	-69%	-95%	-98%
**STB 44**	-75%	-98%	-99%	-100%	-100%
**STB 45**	-54%	-64%	-81%	-94%	-96%
**STB 46**	-44%	-79%	-82%	-93%	-96%
**STB 47**	-21%	-32%	-44%	-84%	-90%
**STB 48**	+34%	+92%	-83%	-92%	-97%
**STB 49**	-17%	-24%	-22%	-90%	-95%
**STB 50**	+101%	-65%	-70%	-92%	-95%

Table S2C. (increase/decrease) percent (%) of growth compared to the control.

Isolate No.	3% NaCl	6% NaCl	9% NaCl	12% NaCl	15% NaCl
**STB 51**	-24%	-57%	-76%	-92%	-97%
**STB 52**	-18%	-56%	-70%	-92%	-99%
**STB 53**	+13%	-6%	-55%	-93%	-94%
**STB 54**	+33%	-38%	-77%	-91%	-96%
**STB 55**	-65%	-74%	-86%	-95%	-97%
**STB 56**	-54%	-68%	-74%	-92%	-97%
**STB 57**	-26%	-41%	-77%	-88%	-92%
**STB 58**	-55%	-67%	-92%	-97%	-99%
**STB 59**	+58%	+30%	-30%	-68%	-28%
**STB 60**	+79%	+44%	-15%	-69%	-78%
**STB 61**	+59%	-71%	-83%	-95%	-98%
**STB 62**	+9%	-29%	-83%	-88%	-93%
**STB 63**	+38%	+79%	+2%	-81%	-89%
**STB 64**	+60%	+18%	-72%	-84%	-97%
**STB 65**	+9%	-29%	-60%	-88%	-96%
**STB 66**	-61%	-63%	-85%	-86%	-98%
**STB 67**	+18%	-15%	-26%	-92%	-99%
**STB 68**	-56%	-61%	-69%	-94%	-96%
**STB 69**	-47%	-61%	-69%	-93%	-98%
**STB 70**	-19%	-36%	-39%	-80%	-84%
**STB 71**	-36%	-60%	-86%	-94%	-98%
**STB 72**	-55%	-74%	-84%	-93%	-97%
**STB 73**	-20%	-54%	-93%	-98%	-100%
**STB 74**	-42%	-54%	-71%	-76%	-83%
**STB 75**	-19%	-36%	-42%	-51%	-89%

Table S2D. (increase/decrease) percent (%) of growth compared to the control.

Isolate No.	3% NaCl	6% NaCl	9% NaCl	12% NaCl	15% NaCl
**STB 76**	-41%	-48%	-88%	-94%	-94%
**STB 77**	-19%	-30%	-32%	-55%	-75%
**STB 78**	-53%	-59%	-69%	-86%	-95%
**STB 79**	-26%	-35%	-55%	-87%	-89%
**STB 80**	-54%	-62%	-67%	-78%	-90%
**STB 81**	-57%	-67%	-76%	-83%	-95%
**STB 82**	-12%	-44%	-64%	-88%	-90%
**STB 83**	-6%	-28%	-45%	-79%	-90%
**STB 84**	-33%	-42%	-84%	-92%	-95%
**STB 85**	-18%	-32%	-49%	-75%	-82%
**STB 86**	+16%	-46%	-48%	-70%	-96%
**STB 87**	-21%	-47%	-72%	-94%	-96%
**STB 88**	-14%	-14%	-26%	-50%	-62%
**STB 89**	+32%	-14%	-25%	-41%	-63%
**STB 90**	+80%	-24%	-31%	-86%	-93%
**STB 91**	-30%	-49%	-96%	-96%	-97%
**STB 92**	-46%	-79%	-87%	-96%	-98%
**STB 93**	-64%	-82%	-88%	-91%	-93%
**STB 94**	-60%	-75%	-93%	-93%	-96%
**STB 95**	-8%	-42%	-62%	-76%	-95%
**STB 96**	-28%	-53%	-89%	-90%	-93%
**STB 97**	-24%	-48%	-91%	-98%	-99%

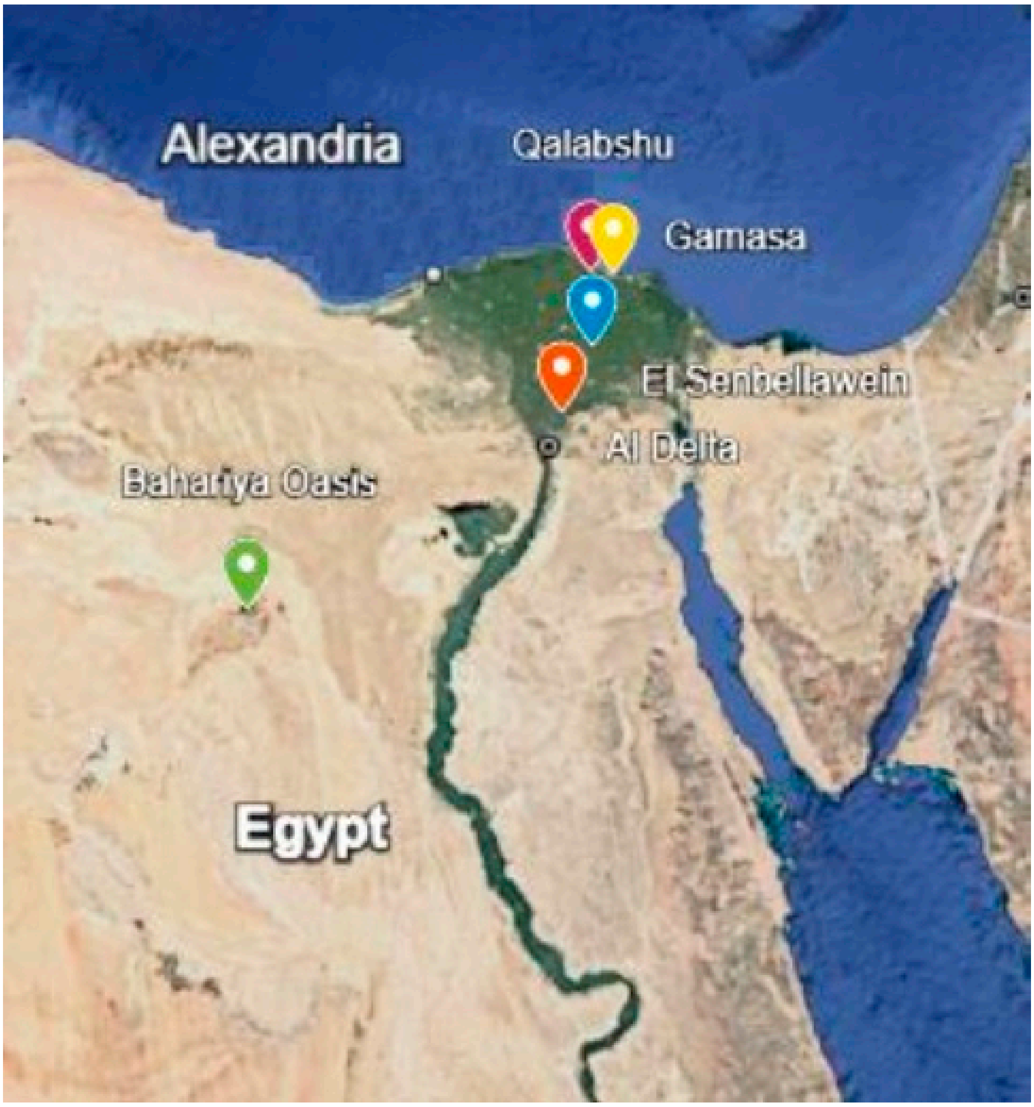

Figure S1. A map showing the locations from where soil samples were collected

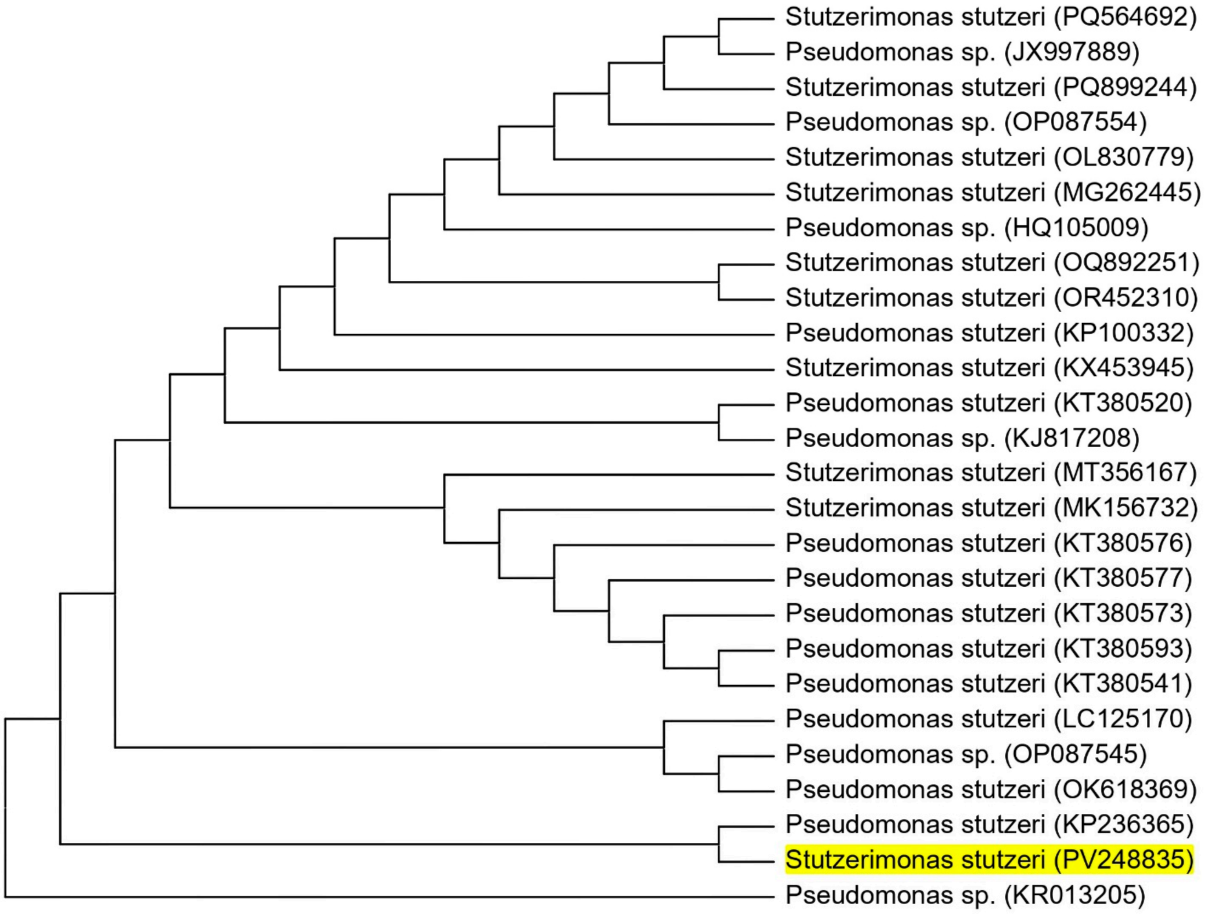

Figure S2. Phylogenetic tree of *Stutzerimonas stutzeri* PV248835
